# Neutrophil fate in bone infection: Mechanisms, microenvironment, and therapeutic opportunities

**DOI:** 10.1016/j.jot.2026.101232

**Published:** 2026-10-24

**Authors:** Chenguang Cai, Yu Ji, Jiawei Mei, Zhengxi Wang, Quan Liu, Wangyang Fan, Yiming Zhao, Xiang Chen, Chen Zhu, Wanbo Zhu, Qianming Li

**Affiliations:** a Graduate School of Bengbu Medical University, Bengbu, China; b Department of Orthopedics, The First Affiliated Hospital of USTC, Division of Life Sciences and Medicine, University of Science and Technology of China, Hefei, Anhui, China; c Department of Orthopedics, Shannan People's Hospital, Shannan, Tibet, China

**Keywords:** Biofilm, Bone infection, Neutrophil, Neutrophil extracellular traps, Orthopaedic infection, Osteomyelitis

## Abstract

The clinical challenge of eradicating bone infection stems from microbial persistence within a distinct skeletal niche that is poorly perfused, hypoxic, mineralised, and highly susceptible to collateral damage. Neutrophils are central arbiters of this process and function as a double-edged sword: they are indispensable for initial pathogen containment, yet can become major drivers of chronic osteolysis, biofilm persistence, and inflammation when their effector programmes turn maladaptive. Recent high-resolution single-cell and spatial analyses have overturned the traditional view of neutrophils as short-lived, homogeneous cells, instead revealing a complex spectrum of functional states shaped by the infected bone microenvironment. In this Review, we use a neutrophil fate framework that distinguishes three related but non-equivalent levels: irreversible terminal fates, non-terminal tissue-disposition states and outcomes, and upstream fate-biasing states or processes. Terminal fates include apoptosis with subsequent efferocytic clearance, suicidal NETosis, pyroptosis, necroptosis, ferroptosis, mitochondrial permeability transition-driven necrosis, and secondary necrosis. By contrast, non-lytic (vital) NET release is considered an effector process rather than a terminal fate. Reverse migration is treated as a non-terminal tissue-exit outcome, whereas inflammatory retention is considered a non-terminal persistence state. Upstream fate-biasing factors include phenotypic or activation states, such as cellular ageing, NET-prone activation, and apoptosis delay, as well as regulatory processes including autophagy and metabolic or redox stress; none of these is classified as an independent terminal fate. We further examine how these terminal fates, non-terminal tissue-disposition states and outcomes, and upstream fate-biasing states and processes are spatially organised across osteomyelitic lesions and biofilm-associated interfaces, and how they govern interactions with osteoblasts, osteoclasts, and macrophages to determine the balance between antimicrobial control and progressive skeletal destruction. We also discuss emerging therapeutic paradigms aimed not at broadly suppressing neutrophil recruitment, but at precisely redirecting neutrophil fate, thereby providing a conceptual basis for stage-specific immunomodulation in orthopaedic infection.

The Translational Potential of this Article

This Review provides a fate-based framework for understanding how neutrophils shape the balance between pathogen control, biofilm persistence, osteolysis, and tissue repair in orthopaedic infection. By linking neutrophil fate programmes to distinct lesion microenvironments, including osteomyelitic lesions, implant-associated biofilms, contiguous soft-tissue infection, and fracture-related infection, this article highlights potential translational opportunities for stage- and compartment-specific immune modulation. These include improving patient stratification, guiding the timing of surgical source control and implant retention or removal, optimising dead-space management and local antimicrobial delivery, supporting vascularised soft-tissue reconstruction, and developing biomarkers that distinguish protective antimicrobial neutrophil responses from destructive inflammatory persistence. This framework may help shift future orthopaedic infection management from nonspecific suppression of inflammation toward precision redirection of maladaptive neutrophil fate while preserving early host defence and tissue repair capacity.

## Graphical abstract

**Figure:**
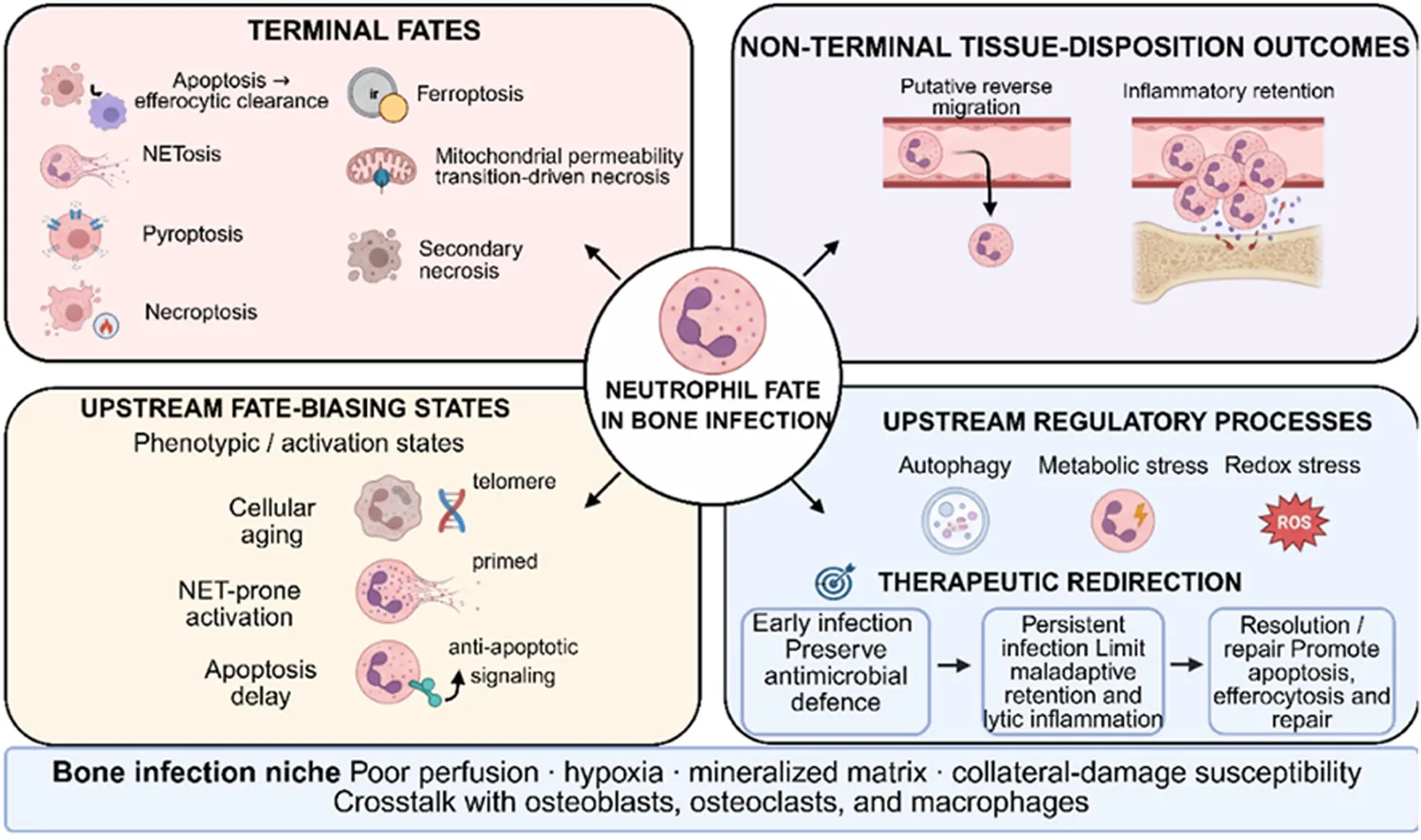

## 1 Introduction

Bone infection, including osteomyelitis, fracture-related infection, and periprosthetic joint infection, remains a major challenge in orthopaedic surgery. These disorders are defined not only by bacterial burden but also by a tissue context marked by low perfusion, hypoxia, mineralised matrix, implant surfaces, and repeated cycles of inflammation and repair [1,2]. *Staphylococcus aureus* (*S*. *aureus*) is the dominant pathogen in most settings, although polymicrobial infection, Gram-negative pathogens, and fungal coinfection may also occur in selected clinical contexts [3,4]. Across these disease entities, persistence is promoted by intracellular survival, abscess formation, biofilm maturation, and poor antimicrobial penetration [[5], [6], [7], [8], [9]].

The central problem is therefore not simple failure of pathogen killing. Rather, bone infection is sustained by reciprocal amplification between microbes and host tissue [8,10]. Biofilms and damaged tissue release pathogen-associated and damage-associated signals that recruit neutrophils and other myeloid cells. In turn, neutrophil proteases, reactive oxygen species, histones, cytokines, and extracellular traps can damage osteoblasts, support osteoclastogenesis, and destabilise osseointegration [1,[11], [12], [13], [14]]. This osteoimmune loop is one reason why bacterial persistence and skeletal destruction often progress together.

This Review uses a three-level neutrophil fate framework (Fig. 1). Terminal fates are defined as irreversible death or terminal-clearance outcomes, including apoptosis with subsequent efferocytic clearance, suicidal NETosis, pyroptosis, necroptosis, ferroptosis, mitochondrial permeability transition-driven necrosis, and secondary necrosis [[15], [16], [17]]. Non-lytic (vital) NET release is distinguished from suicidal NETosis and is treated as an effector process rather than a terminal fate. Non-terminal tissue-disposition states and outcomes include inflammatory retention and reverse migration, respectively. Upstream fate-biasing factors include phenotypic or activation states, such as cellular ageing, N1/N2-like polarisation, NET-prone activation, and apoptosis delay, as well as regulatory processes and microenvironmental pressures including autophagy, hypoxia, acidosis, redox stress, iron dysregulation, and metabolic exhaustion [[16], [17], [18], [19]]. These three levels are conceptually distinct and are used consistently throughout this Review.

**Fig. 1:**
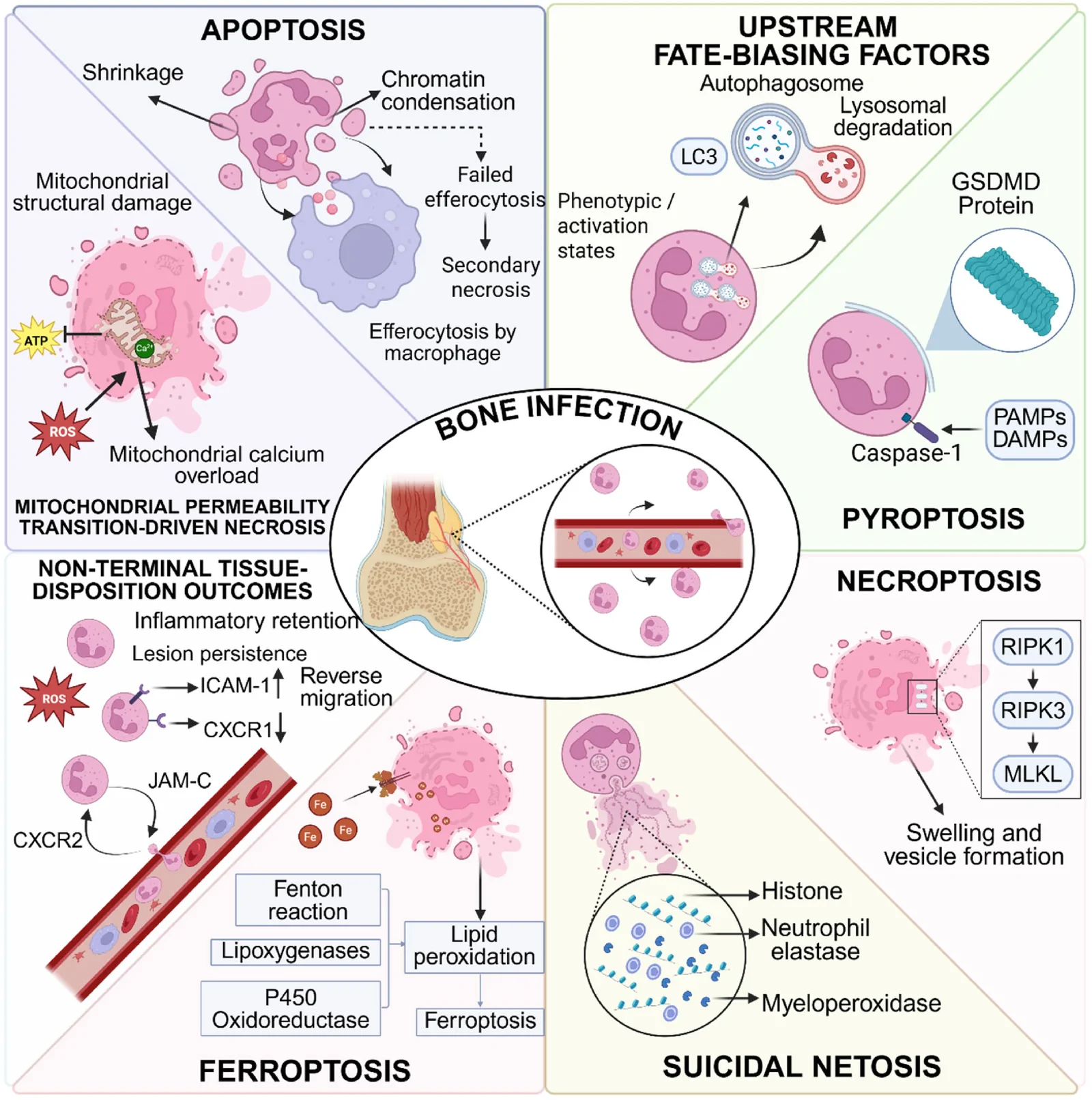
| Neutrophil terminal fates, non-terminal tissue-disposition states and outcomes, and upstream fate-biasing factors in bone infection.

These categories also require distinct evidentiary criteria. Terminal fates should be assigned using pathway-linked structural, biochemical, and functional readouts, whereas reverse migration requires evidence of tissue exit or vascular re-entry [[15], [16], [17],[19], [20], [21]]. Phenotypic or activation states should be defined using multimarker and functional or omics-based approaches rather than single surface markers, while autophagy and metabolic or microenvironmental stressors are treated as upstream regulatory processes rather than independent fates [18,[21], [22], [23], [24], [25], [26], [27], [28], [29], [30], [31], [32]].

The skeletal niche is therefore best understood as a host-microbe-material ecosystem. Mineralised matrix restricts diffusion. Necrotic bone creates poorly perfused or avascular niches that favour microbial persistence [1,8]. Implants provide non-physiological surfaces that favour early bacterial attachment and matrix maturation [2,9,[15], [16], [17], [18], [19], [20], [21]]. Repeated surgery, instability, and local ischaemia further amplify damage-associated molecular pattern (DAMP) release and alter chemokine gradients. Neutrophils sit at the centre of this ecosystem because they are the first cells to arrive at most infected lesions and because their products immediately reshape the local environment. They can contain spread, instruct other leukocytes, and facilitate abscess organisation [4,22]. They can also injure osteoblasts, potentiate osteoclastogenesis, stabilise matrix-rich biofilm communities, and transmit inflammation systemically when clearance fails [[11], [12], [13],[23], [24], [25], [26]]. A review framed around neutrophil fate can therefore integrate microbiology, osteoimmunology, and tissue repair more effectively than a review organised only around pathogens or only around generic innate immunity. Accordingly, neutrophil fate in infected bone should not be interpreted as a purely cell-intrinsic death programme. It is continuously shaped by macrophage efferocytosis, osteoclastogenic and osteoblast-lineage responses, endothelial activation, platelet-driven thromboinflammation, and biomaterial-associated cues [[17], [18], [19], [20],[27], [28], [29], [30]].

A second reason bone infection remains difficult to treat is that the dominant clinical entities only partially overlap in biology. Haematogenous osteomyelitis, contiguous osteomyelitis, periprosthetic joint infection, and fracture-related infection share the problem of bacterial persistence, but they differ in vascular supply, burden of necrotic tissue, biomaterial exposure, and the degree to which repair programmes remain active. *S. aureus* is still the most common pathogen, yet the lesion-level biology is also shaped by coagulase-negative staphylococci, Gram-negative bacilli, mixed communities, and in selected settings fungi or mycobacteria [3,4,8]. Each of these settings imposes a different pattern of neutrophil recruitment, activation, and clearance [4,31]. The same neutrophil programme that is useful during abrupt bacteraemia or early contamination can become maladaptive when bacteria are sheltered within biofilm, osteocyte-lacuno-canalicular networks, osteoblasts, or macrophages [[5], [6], [7],^9^]^.^

We therefore use neutrophil fate not only as a mechanistic framework, but also as a translational lens through which key orthopaedic decisions, including debridement, implant retention or removal, dead-space management, local antibiotic delivery, soft-tissue coverage, and management of infection-related nonunion, can be reconsidered [21,32].

The figure illustrates three related but distinct levels of neutrophil biology. Terminal fates include apoptosis with subsequent efferocytic clearance and inflammatory death programmes such as suicidal NETosis, pyroptosis, necroptosis, ferroptosis, mitochondrial permeability transition-driven necrosis, and secondary necrosis. Non-lytic (vital) NET release is distinguished from suicidal NETosis and is shown as an effector process rather than a terminal fate. Reverse migration is treated as a non-terminal tissue-disposition outcome involving neutrophil exit from inflamed tissue, whereas inflammatory retention is treated as a non-terminal persistence state within the lesion. Upstream fate-biasing factors include phenotypic or activation states, as well as regulatory processes and microenvironmental pressures. Cellular ageing, N1/N2-like polarisation, NET-prone activation, and immunoregulatory phenotypes are included within this upstream layer but are not depicted as separate compartments. Autophagy, metabolic and redox stress, hypoxia, nutrient limitation, bacterial products, cytokines, and biomaterial-associated cues are shown as representative fate-regulatory inputs rather than independent terminal fates.

Abbreviations: ATP, adenosine triphosphate; CXCR1/2, C-X-C chemokine receptors 1 and 2; DAMPs, damage-associated molecular patterns; GSDMD, gasdermin D; ICAM-1, intercellular adhesion molecule 1; JAM-C, junctional adhesion molecule C; LC3, microtubule-associated protein 1A/1B-light chain 3; MLKL, mixed lineage kinase domain-like protein; PAMPs, pathogen-associated molecular patterns; RIPK1/3, receptor-interacting protein kinases 1 and 3; ROS, reactive oxygen species.

## 2 Evidence hierarchy and inferential boundaries of the spatial models

The spatial models proposed in this Review should be interpreted as conceptual syntheses rather than as direct maps derived from a single human bone-infection dataset. We distinguish three levels of evidence. First, clinical and translational studies of orthopaedic infection directly support several core observations, including neutrophil-rich inflammation in osteomyelitis, NET-associated mediators at infected bone or implant interfaces, impaired immune clearance in biofilm-associated infection, and the association between persistent neutrophil activation and osteolytic tissue damage [11,12,20,25,[33], [34], [35], [36], [37]]. Second, additional relationships are supported primarily by animal models of osteomyelitis, implant infection, fracture-related infection, or diabetic wound infection, in which lesion topology, bacterial burden, hypoxia, and host-cell injury can be examined more directly [[38], [39], [40], [41], [42]]. Third, several zone-specific assignments remain mechanistic extrapolations from related inflammatory, infectious, or wound-healing models. These include the proposed enrichment of necroptosis, ferroptosis, and mitochondrial permeability transition-driven necrosis; reverse migration as a non-terminal tissue-exit outcome; and the localisation of apoptosis-delayed or immunoregulatory neutrophil states [23,[43], [44], [45], [46], [47], [48], [49], [50], [51], [52], [53], [54], [55], [56], [57]]. Reverse migration warrants particular caution because, although it has been demonstrated in other inflammatory and infection-related settings, direct evidence for a substantial role in human osteomyelitis, fracture-related infection, or periprosthetic joint infection remains limited [58,59]. These spatial assignments are therefore used to organise testable hypotheses rather than as definitive maps of human bone infection.

## 3 Neutrophils in bone infection, from terminal effectors to heterogeneous tissue programmes

Neutrophils were historically viewed as short-lived terminal effectors that entered infected tissue, phagocytosed microbes, degranulated, generated reactive oxygen species, and rapidly died [60,61]. That framework remains partly correct for acute infection, but it is now insufficient for chronic skeletal infection. Over the past decade, transcriptomic, proteomic, metabolic, and fate-mapping studies have shown that neutrophils diversify across maturation states, tissue niches, and inflammatory contexts [33,34,[62], [63], [64], [65], [66], [67], [68]]. They can acquire distinct chemotactic, antimicrobial, immunosuppressive, antigen-presenting, reparative, or pathogenic features, and these states are not fully captured by a small number of surface markers [[69], [70], [71], [72], [73], [74], [75], [76], [77], [78]].

This conceptual shift is particularly important in bone infection. Neutrophils that encounter planktonic bacteria, necrotic bone, implant biofilm, diabetic soft tissue, or resolving granulation tissue are unlikely to behave identically [20,31,33,34]. Instead, they integrate local cytokines, stromal signals, vascular cues, and metabolic stress [[18], [19], [20],^31^,^42^,^79^]. The result is a continuum of phenotypes that can include early bactericidal cells, apoptosis-delayed or aged phenotypes that bias neutrophils toward persistent inflammatory retention, NET-prone cells, immunoregulatory subsets, and cells with features consistent with tissue-exit or reverse-migration-like programmes or secondary tissue injury [34,[80], [81], [82], [83], [84], [85], [86], [87]].

### 3.1 Phenotypic polarisation as a fate-biasing state

Neutrophil phenotypic polarisation provides an important bridge between environmental sensing and fate choice in bone infection [31,34,[62], [63], [64], [65], [66], [67], [68],^76^,^88^]. Rather than representing fixed lineages, these context-dependent neutrophil states should be viewed as dynamic functional biases shaped by bacterial products, hypoxia, cytokines, stromal cues, biofilm matrix, and biomaterial-associated signals. In early or well-controlled infection, neutrophils may adopt a predominantly antimicrobial phenotype characterised by chemotaxis, phagocytosis, oxidative burst, degranulation, and limited NET release [60,61,69,89]. In persistent or poorly cleared lesions, the same cells may shift toward NET-prone, apoptosis-delayed, aged CXCR4^hi, inflammasome-linked, or tissue-damaging phenotypes that sustain protease release, cytokine amplification, osteoclastogenic inflammation, and biofilm persistence [11,74,75,79,80,90]. Conversely, under more vascularised and resolution-permissive conditions, neutrophils may acquire immunoregulatory or repair-associated features that support macrophage efferocytosis, angiogenesis, and tissue remodelling [[27], [28], [29], [30],^91^]^.^

Although N1/N2-like terminology has been used in some inflammatory and tumour contexts, this binary classification may oversimplify neutrophil behaviour in skeletal infection [[65], [66], [67],^70^,^77^,^78^,^88^]. Bone infection is spatially heterogeneous, and neutrophils exposed to necrotic bone, implant biofilm, ischaemic soft tissue, or reparative marrow are unlikely to occupy a single polarised endpoint. We therefore discuss neutrophil polarisation as a reversible and context-dependent state that biases subsequent states or outcomes, including apoptosis, suicidal NETosis, inflammatory retention, reverse migration, secondary necrosis, or resolution, rather than as an independent fate category [30,68,91,92]. For hypothesis-generating phenotyping, candidate marker combinations associated with major neutrophil states in mouse models and human orthopaedic infection samples are summarised in Table 1 [74,75]. These combinations are not sufficiently specific for standalone state assignment and require functional and spatial validation.

**Table 1:** | Candidate marker combinations for phenotyping neutrophil states in mouse models and human orthopaedic infection samples.

Candidate phenotype/state	Candidate mouse markers	Candidate human markers	Possible interpretation	Required validation/caveat
Core neutrophil gating	CD45^+^ Ly6G^+^ CD11b^+^; often Ly6C^int^	CD45^+^ CD15^+^ CD66b^+^ CD11b^+^; CD16 variable	Establishes a neutrophil-enriched gate for further phenotyping	Use viability dye and lineage exclusion; avoid relying on one marker
Recently recruited/activated candidates	Ly6G^+^ CD11b^hi^ CXCR2^+^ CD62L^hi/int^	CD66b^+^ CD11b^hi^ CD62L^hi/int^ CD16^hi^	May indicate recent recruitment or activation	Recruitment, activation, and maturation overlap; confirm by function and tissue context
Ageing-/retention-associated candidates	Ly6G^+^ CXCR4^^hi^ CD62L^lo^ CD11b^hi^	CD66b^+^ CXCR4^^hi^ CD62L^lo^ CD11b^hi^	May indicate ageing, retention, or delayed-clearance programmes	CXCR4/CD62L are not specific for ageing or apoptosis delay; require functional and spatial validation
NET-prone/highly inflammatory candidates	Ly6G^+^ CD11b^hi^; MPO, NE, citH3 or PAD4-associated readouts	CD66b^+^ CD11b^hi^; MPO, NE, citH3 or PAD4-associated readouts	May indicate NET-associated inflammatory potential	Cannot distinguish suicidal NETosis from non-lytic NET release; requires functional/imaging confirmation
Immunoregulatory/suppressive-like candidates	Ly6G^+^ CD11b^+^ Arg1^+^ PD-L1^+^; sometimes CD62L^lo^	CD66b^+^ CD11b^+^ ARG1^+^ PD-L1^+^; CD16^lo^ or low-density fraction in selected settings	May indicate immunoregulatory potential	Marker expression alone does not establish suppressive function; requires functional T-cell assays and spatial validation
PLAUR/CD87-associated candidates	Ly6G^+^ CD11b^+^ CD87/PLAUR^+^	CD66b^+^ CD11b^+^ CD87/PLAUR^+^	May indicate disease-associated activation or remodelling	Context-dependent and non-specific; direct orthopaedic-infection validation remains limited
CD177+ activated candidates	Ly6G^+^ CD11b^+^ CD177^+^ if available	CD66b^+^ CD177^+^	May indicate an activated or functionally distinct subset	CD177 varies markedly among individuals and does not define a unique functional state
Tissue-interacting/reverse-migration-like candidates	Ly6G^+^ ICAM-1^hi^ CXCR1/2 altered; CD62L^lo^	CD66b^+^ ICAM-1^hi^ CXCR1/2 altered; CD62L^lo^	May indicate altered endothelial interaction or trafficking	Markers cannot establish reverse migration; requires direct spatial, lineage-tracing, or vascular re-entry evidence

**Abbreviations:** Arg1/ARG1, arginase 1; CD, cluster of differentiation; citH3, citrullinated histone H3; CXCR, C-X-C chemokine receptor; ICAM-1, intercellular adhesion molecule 1; MPO, myeloperoxidase; NE, neutrophil elastase; PAD4, protein arginine deiminase 4; PD-L1, programmed death-ligand 1; PLAUR/CD87, plasminogen activator, urokinase receptor.

These candidate marker combinations should be interpreted as hypothesis-generating phenotyping tools rather than definitive identifiers of neutrophil states. Because activation, maturation, ageing, trafficking, and immunoregulatory programmes can share overlapping surface-marker profiles, marker-based phenotyping alone is insufficient for state assignment. Functional assays, transcriptomic or proteomic profiling, and spatial localisation should therefore be incorporated wherever possible, with direct imaging or lineage-tracing evidence required for dynamic processes such as reverse migration [64,67,72].

A practical implication is that neutrophils should not be considered uniformly beneficial or harmful in bone infection. Their overall effect depends on which state dominates, when it emerges, and where it is located within the lesion [[27], [28], [29],^39^,^91^,[93], [94], [95], [96]]. This is why broad neutrophil depletion has limited translational appeal, whereas selective redirection of neutrophil fate may offer a more realistic therapeutic strategy.

Methodologically, the field has also moved beyond bulk measurements of total neutrophil number or generic activation markers. Single-cell RNA sequencing, spatial transcriptomics, high-parameter cytometry, proteomic profiling, and intravital or ex vivo imaging now allow investigators to map where specific neutrophil states accumulate and which neighbouring cells sustain them [15,33,63,64]. These approaches have been especially informative in chronic inflammatory tissues where local stromal cells, endothelial cells, macrophages, and lymphocytes create stable niches for neutrophil persistence [[48], [49], [50], [51],^70^,^72^]. For bone infection, this is important because lesion sampling is often spatially biased [48]. Tissue collected from purulent exudate, sequestrum-associated interface, membrane around an implant, or healing callus will not report the same biology [48]. Future studies need to pair neutrophil phenotyping with lesion topology, microbial architecture, and clinical stage [68,97]. Without that integration, the field risks describing heterogeneity without identifying the states that are truly actionable in orthopaedic infection [66].

This broader view of neutrophil biology has several implications for skeletal infection. First, heterogeneity begins before tissue entry. Bone marrow output during infection includes mature segmented neutrophils, recently released immature forms, and stress-associated populations with altered transcriptional programmes [62,63,88]. Once in circulation, circadian ageing, microbiome-derived signals, systemic cytokines, and metabolic disease can further tune their responsiveness [69,85,86,98]. After tissue entry, endothelial cues, platelet interactions, stromal ligands, and microbial products act on top of this pre-existing diversity [23,74,83,99]. The result is that lesion neutrophils should be considered state-dependent cells that occupy a moving spectrum rather than a single lineage-locked endpoint [65,78]. This helps explain why aged CXCR4^hi cells, low-density immunoregulatory neutrophils, NET-prone cells, and interferon- or inflammasome-linked states can coexist in the same patient yet contribute very differently to bacterial control and tissue damage [71,[73], [74], [75],^80^].

### 3.2 Bone infection and emergency neutrophil development

Bone infection may also reshape neutrophil origin and development before the cells enter the infected lesion. Because the bone marrow is both the main site of granulopoiesis and, in osteomyelitis, part of the affected tissue environment, local and systemic inflammatory signals can alter neutrophil production, maturation, and release. Bacterial products, damage-associated molecular patterns (DAMPs), IL-1 family cytokines, IL-6, G-CSF, GM-CSF, TNF-α, and IL-17-related pathways may promote emergency granulopoiesis and accelerate the release of immature or stress-associated neutrophils into the circulation [62,69,100,101]. These newly generated or prematurely released cells may differ from homeostatic neutrophils in chemotaxis, oxidative metabolism, degranulation, NET-forming capacity, apoptosis sensitivity, and immunoregulatory potential [71]. In this way, bone infection does not merely recruit pre-existing neutrophils; it may also feed back on the marrow compartment to generate neutrophil populations that are already developmentally biased toward persistence, inflammatory amplification, or impaired resolution. Direct evidence defining these developmental trajectories in human osteomyelitis and implant-associated infection remains limited, and future studies should combine bone marrow, blood, and lesion-level profiling to determine how infection-induced granulopoiesis shapes neutrophil fate in skeletal disease [33,42].

### 3.3 Pathogen-specific modulation of neutrophil fate

Bone infection should not be treated as a microbiologically uniform entity, because different pathogens can bias neutrophil fate through distinct virulence factors, biofilm architectures, and immune-evasion strategies. *S. aureus*, the dominant pathogen in many forms of osteomyelitis and implant-associated infection, can directly manipulate neutrophil survival and lytic death through pore-forming toxins and leukocidins, including α-hemolysin, Panton-Valentine leukocidin, phenol-soluble modulins, and related cytolytic factors [3,4,26,79,89,102]. These signals can promote membrane injury, inflammasome activation, NET release, or secondary necrosis, while staphylococcal nucleases and biofilm matrix components may degrade or remodel NETs into structures that support bacterial persistence rather than clearance [7,9,[102], [103], [104]]. Thus, *S. aureus* infection may generate a neutrophil fate pattern characterised by intense early recruitment, abscess formation, toxin-driven lytic injury, NET remodelling, and persistent osteolytic inflammation [11,13,22,25].

By contrast, Gram-negative biofilm-associated pathogens, such as *Pseudomonas aeruginosa* and Enterobacteriaceae, may shape neutrophil fate through different mechanisms. Lipopolysaccharide (LPS), flagellin, type III secretion systems, redox-active metabolites, proteases, extracellular polymeric substances, and bacterial outer membrane vesicles (OMVs) can sustain neutrophil activation, oxidative stress, delayed clearance, inflammasome-linked signalling, and persistent NET formation [31,32,87,105]. In mature Gram-negative biofilms, neutrophils may remain abundant but ineffective, contributing extracellular DNA, proteases, and oxidants to a matrix-rich niche that favours immune evasion and antibiotic tolerance [41,[103], [104], [105], [106]].

Coagulase-negative staphylococci may produce less acute cytolytic injury than *S. aureus*, but their implant-associated biofilms can still promote frustrated phagocytosis, delayed neutrophil turnover, and chronic low-grade NET-associated inflammation [90,[107], [108], [109]]. Anaerobe-rich and polymicrobial infections, particularly in diabetic or contiguous soft-tissue wounds, may further amplify neutrophil dysfunction through hypoxia, protease accumulation, nutrient limitation, short-chain fatty acid metabolites, and mixed PAMP-DAMP stimulation [[110], [111], [112]]. Fungal co-infections, although less common, may also alter neutrophil fate through β-glucan- and mannoprotein-driven pattern-recognition signalling, impaired phagocytic clearance, persistent oxidative activation, and NET-associated fungal containment [89]. These non-*S. aureus* pathogens therefore broaden the fate landscape from acute toxin-driven lytic injury toward chronic biofilm-associated retention, persistent NET release and accumulation, defective efferocytosis, and metabolically constrained neutrophil dysfunction [30,36,73]. Recent translational evidence further indicates that host metabolic conditions can directly reshape bacterial behaviour at implant interfaces: in diabetic periprosthetic joint infection, synovial advanced glycosylation end products promoted *S. aureus* RNAIII expression and biofilm formation [113].

These pathogen-specific differences have practical implications for neutrophil-directed therapy. Interventions designed to limit excessive NET formation, block inflammasome signalling, modulate redox stress, or engineer immune-active biomaterials may perform differently depending on whether the dominant microbial driver is toxin-producing *S. aureus*, matrix-protected coagulase-negative staphylococci, Gram-negative biofilm-forming organisms, or polymicrobial wound flora [32,36,102]. Therefore, future studies should pair neutrophil fate profiling with pathogen identification, virulence-factor analysis, and biofilm characterisation, rather than interpreting all orthopaedic infections through a single neutrophil response model [97].

## 4 The double-edged role of neutrophils in osteoimmunity

Within infected bone, neutrophils sit at the interface of antimicrobial defence and skeletal remodelling. Early recruitment is essential for containment of invasive bacteria and for construction of an organised abscess that limits dissemination [22,60,89]. Efficient pathogen clearance followed by apoptosis and efferocytosis can favour macrophage reprogramming, reduce inflammatory tone, and create conditions that are permissive for tissue repair [27,28,30,91,93].

The same cells can also drive bone loss. Neutrophil-derived IL-1β, S100A8/A9, proteases, oxidants, histones, and NET-associated proteins amplify local inflammation and can directly or indirectly promote osteoclast differentiation and osteoclast activity [13,14,25,114]. Clinical and translational studies in implant-associated inflammation support this connection, showing enrichment of neutrophil activation programmes at osteolytic interfaces and linking persistent neutrophil activation to loosening and failed osseointegration [12,25,35].

The key point is that osteoimmune coupling is not disrupted only by bacteria. It is also reshaped by the fate choice of infiltrating neutrophils. A lesion dominated by efficient early clearance and timely efferocytosis is biologically different from a lesion dominated by delayed apoptosis, NET accumulation, and secondary necrosis [[91], [92], [93],^115^,^116^]. Any review of bone infection therefore needs to consider fate as a central determinant of both pathogen control and bone outcome [12,13].

The flip side is that appropriate neutrophil turnover can support repair. When pathogen burden falls and neutrophils undergo timely apoptosis, macrophage efferocytosis drives production of IL-10, TGF-β, and other pro-resolving mediators [27,28]. This switch lowers inflammatory tone, limits further leukocyte influx, and opens a window for angiogenesis and osteogenesis [29]. Neutrophils may also contribute indirectly to regeneration by releasing extracellular vesicles, annexin-linked mediators, or growth-supportive signals before they are cleared [29,84,117]. These reparative effects are easy to overlook in chronic infection because destructive states dominate clinically, but they matter for translation. The therapeutic goal is not to remove neutrophils from infected bone. It is to preserve early antibacterial competence while accelerating the transition toward a resolution-competent niche that permits bone healing and osseointegration [39,[93], [94], [95]].

In practical terms, neutrophils influence bone by at least four nonexclusive routes. They release inflammatory mediators, including IL-1 family cytokines and the alarmins S100A8/A9, that promote osteoclastogenic inflammation [69,118]. They discharge proteases that degrade extracellular matrix and expose new DAMPs [89,115,116,119]. They generate oxidants and NET-associated histones that are directly cytotoxic to stromal and endothelial cells [23,24,26,119]. They also shape the behaviour of monocytes and macrophages, which in turn control the balance between osteoclast formation and repair. This network means that bone loss in infection is not simply a consequence of bacterial toxins. It is also a systems-level output of unresolved neutrophilic inflammation [1,120]. Implant loosening and cortical destruction are therefore best viewed as emergent properties of persistent host-microbe crosstalk rather than purely microbial invasion [35].

### 4.1 Cellular, adaptive-immune, and material crosstalk shaping neutrophil fate

Neutrophil fate in bone infection is shaped by reciprocal interactions with resident and recruited cells, as well as by biomaterial surfaces. Macrophages are central partners because they determine whether apoptotic neutrophils are cleared through efferocytosis or progress to secondary necrosis [116]. Neutrophil-derived NET components, reactive oxygen species (ROS), S100 proteins, proteases, and IL-1 family mediators can injure osteoblast-lineage cells, impair matrix formation, and promote osteoclastogenic inflammation, thereby coupling persistent neutrophilic inflammation to osteolysis and failed osseointegration [14,23].

Endothelial cells and platelets provide additional regulatory interfaces. Endothelial activation controls neutrophil recruitment and tissue entry, whereas excessive NET formation, proteases, and oxidants can damage the microvasculature, promote immunothrombosis, and aggravate local hypoxia [24,26,58,119]. Platelets may further amplify NET formation and thromboinflammatory remodelling at infected or biofilm-associated interfaces. Biomaterials add a non-cellular but highly relevant fate-selecting surface: implant topography, protein adsorption, wear particles, corrosion products, micromotion, and bacterial biofilm can promote neutrophil adhesion, frustrated phagocytosis, delayed clearance, and NET-rich inflammatory retention [18,19,21,36,42,90]. Thus, neutrophil fate in orthopaedic infection should be understood as an output of multicellular and material-dependent crosstalk rather than as a purely autonomous death programme.

Neutrophils may also connect the early innate response to adaptive immunity in bone infection. Beyond direct bacterial killing, activated or aged neutrophils can release cytokines, chemokines, NET-associated molecules, and antigen-presenting signals that influence T-cell recruitment, activation, differentiation, and effector function [70,80,121]. In parallel, neutrophil-derived mediators, including BAFF, APRIL, and inflammatory cytokines, may support B-cell survival, plasma-cell differentiation, antibody production, and local immune memory. These adaptive immune effects may be beneficial when they strengthen pathogen containment and limit dissemination, but they may become harmful when persistent neutrophil activation sustains excessive T-cell and B-cell responses, chronic inflammation, and osteolytic tissue damage [80,121]. Direct spatial evidence for neutrophil-driven T- and B-cell regulation in human bone infection remains limited, and this axis should therefore be considered an important area for future osteoimmunology studies.

## 5 Resolution-associated clearance and non-terminal tissue exit: apoptosis, efferocytosis, and reverse migration

This section distinguishes resolution-associated terminal clearance through apoptosis and subsequent efferocytosis from reverse migration, a non-terminal tissue-exit outcome whose consequences may include either reduced local neutrophil burden or systemic inflammatory spillover. Autophagy is discussed separately as an upstream fate-regulatory process rather than as an independent fate. Apoptosis remains the canonical mechanism by which neutrophils are removed without spilling toxic intracellular contents [93,[122], [123], [124], [125]]. In inflamed tissue, successful execution of apoptosis, followed by macrophage efferocytosis, limits further leukocyte recruitment and supports a shift toward anti-inflammatory cytokine production and repair-associated macrophage states. In this context, timely neutrophil apoptosis followed by efficient efferocytosis constitutes a key component of inflammatory resolution, limiting further tissue injury once microbial control has been established [85,116,123].

Bone infection often interferes with this transition. Survival cues such as GM-CSF, IL-6, PI3K signalling, MAPK activation, and myeloid cell leukaemia 1 (Mcl-1) stabilisation can delay apoptosis [[124], [125], [126], [127], [128], [129], [130], [131]]. When clearance is delayed, neutrophils remain metabolically active long enough to continue releasing proteases, oxidants, and osteoclastogenic mediators. This can convert a protective acute infiltrate into a chronic destructive compartment [[132], [133], [134]].

Reverse migration is considered separately from apoptosis and efferocytosis because it represents a non-terminal tissue-exit outcome rather than an intrinsically pro-resolving programme. Its relevance to bone infection remains potentially important but indirectly supported. In non-skeletal inflammatory and infection-related models, some neutrophils can leave inflamed tissue and re-enter the circulation rather than dying locally [59,135]. This process may reduce local neutrophil burden, but reversely migrated neutrophils may also retain an activated phenotype and contribute to systemic inflammatory injury [58]. In bone infection, direct evidence for reverse migration remains limited, and its relevance should therefore be considered inferential. It may be most plausible in vascularised inflammatory margins rather than in necrotic bone, mature biofilm, or poorly perfused sequestrum-associated compartments. Future spatial and lineage-tracing studies are needed to determine whether reverse migration contributes meaningfully to resolution, systemic spillover, or persistent inflammation in osteomyelitis and implant-associated infection [58,59,135].

Autophagy provides an upstream fate-regulatory layer that may support neutrophil homeostasis and controlled clearance but can also alter NET formation and inflammatory death-pathway selection. Basal autophagy supports homeostasis, and stress-induced autophagy can preserve ATP supply, remove damaged organelles, and restrain excessive inflammatory output [79,115,117,136]. In bone infection, this pathway is relevant because neutrophils often experience nutrient limitation, hypoxia, and toxic ROS exposure [117]. A modest autophagic response may help maintain antibacterial function and prevent uncontrolled lytic death. By contrast, defective autophagy can trap neutrophils in an immature or dysfunctional state, whereas excessive or dysregulated autophagy may intersect with cell death pathways and alter NET formation [56,92,115,136]. This makes autophagy less a single therapeutic target than a regulator of whether a stressed neutrophil retains controlled antimicrobial function, undergoes ordered apoptosis and clearance, or progresses towards inflammatory collapse [25,[124], [125], [126], [127], [128], [129], [130],^132^]. In this Review, autophagy is therefore treated as a fate-regulatory process rather than as an independent neutrophil fate [117].

Efferocytosis is equally important because apoptosis is protective only if clearance follows. Macrophages that ingest apoptotic neutrophils are reprogrammed away from inflammatory cytokine output and toward tissue maintenance. If efferocytosis is inefficient, apoptotic cells progress to secondary necrosis and the microenvironment is flooded again with histones, proteases, oxidised lipids, and mitochondrial DAMPs [116,119]. Thus, local inflammatory resolution depends on timely neutrophil apoptosis followed by efficient macrophage efferocytosis. Reverse migration, by contrast, represents a distinct non-terminal tissue-disposition outcome involving neutrophil exit from inflamed tissue rather than local clearance.

## 6 Pro-inflammatory lytic programmes: pyroptosis, necroptosis, and suicidal NETosis

Pyroptosis, necroptosis, and suicidal NETosis are particularly relevant in bone infection because each can amplify antimicrobial alarm signals while also exposing surrounding tissue to substantial collateral damage [92]. These pathways are triggered by distinct upstream mechanisms, but they share a common outcome in chronic lesions, namely release of inflammatory mediators and structural damage to the skeletal niche [35,38].

Pyroptosis is driven by inflammasome signalling and gasdermin-dependent membrane permeabilisation. In neutrophils, this pathway is more plastic than in macrophages and can involve canonical caspase-1 signalling, non-canonical sensing of cytosolic lipopolysaccharide, and protease-dependent cleavage of gasdermin family members [56]. In orthopaedic infection, pyroptosis is most relevant when neutrophils encounter intracellular bacteria, bacterial toxins, ATP, extracellular DNA, or other inflammasome-activating signals within hypoxic and damaged tissue [38,137]. A moderate degree of inflammasome activity may amplify host defence, but persistent activation can sustain IL-1 signalling, accelerate osteoclastogenic inflammation, and promote gasdermin-dependent membrane permeabilisation and lytic cell death. Pyroptosis should therefore be viewed as part of a broader inflammatory circuit rather than as an isolated cell-death programme. In bone infection, it is particularly relevant as an IL-1β- and IL-18-releasing inflammatory programme that may link inflammasome activation to stromal injury and inflammatory bone loss.

Necroptosis becomes important when apoptotic execution is blocked or when death-receptor signalling, bacterial products, hypoxia, or oxidative stress prevent orderly neutrophil clearance. Caspase-8 sits at a critical checkpoint, and inhibition of this axis can permit receptor-interacting protein kinase 1 (RIPK1), receptor-interacting protein kinase 3 (RIPK3), and mixed lineage kinase domain-like protein (MLKL) activation [56,137]. Necroptotic membrane rupture releases HMGB1, IL-1α, proteases, membrane debris, and other DAMPs, which may accumulate in poorly drained skeletal lesions and amplify local inflammation. These products may activate macrophage and stromal nuclear factor kappa B (NF-κB) signalling, reinforcing IL-6- and TNF-α-rich osteolytic inflammation [114,138]. At the same time, complete suppression of necroptotic signalling may not always be desirable, because some necroptosis-related pathways can support host defence under acute conditions [137]. Accordingly, the therapeutic consequences of modulating necroptotic signalling are likely to depend on disease stage and lesion context rather than on assuming that the pathway is uniformly detrimental.

Suicidal NETosis is a terminal lytic programme in which NET release is coupled to irreversible neutrophil death. Classical suicidal NETosis involves chromatin decondensation, PAD4-dependent histone citrullination in many settings, granular enzyme redistribution, strong oxidant signalling, and eventual loss of membrane integrity. By contrast, non-lytic (vital) NET release can occur without immediate cell death and is therefore treated in this Review as an effector process rather than a terminal fate [24,79,81,82,119,132,139,140]. Both routes can generate extracellular NET material capable of immobilising bacteria and concentrating antimicrobial proteins, but prolonged or excessive NET accumulation can expose histones, proteases, oxidised DNA, and other cytotoxic material to bone surfaces and implant interfaces [36,37,90,[107], [108], [109]].

These inflammatory and lytic programmes should be viewed as an interacting network rather than isolated processes. Pyroptotic signalling, necroptotic execution, suicidal NETosis or non-lytic NET release, ferroptotic injury, and broader PANoptosis-like stress responses may coexist under extreme inflammatory pressure [56]. This integrated perspective is important because pyroptotic, necroptotic, ferroptotic, and mitochondrial-collapse pathways may collectively determine whether neutrophil-rich inflammation progresses toward cytokine release, DAMP amplification, or irreversible metabolic failure. Such overlap is relevant in orthopaedic infection because lesions are often exposed simultaneously to bacterial toxins, necrotic debris, hypoxia, and repeated mechanical injury [35,45]. A neutrophil in this environment may not follow a textbook pathway from beginning to end. Instead, it may move through hybrid states that converge on membrane damage, cytokine release, and extracellular deposition of toxic material [24]. Translationally, this means that targeting one node may be insufficient unless the tissue context that drives pathway switching is also corrected [39,94,141].

### 6.1 Molecular switches and regulatory locks linking neutrophil fate programmes

The boundaries between apoptosis, pyroptosis, necroptosis, suicidal NETosis, and stress-driven necrosis are controlled by shared intracellular switches rather than by completely independent pathways. Caspase-8 is a central example. When caspase-8 activity is preserved, death-receptor and inflammatory signalling can still terminate through apoptosis and non-lytic clearance. When caspase-8 activity is inhibited or overwhelmed by bacterial toxins, DAMPs, hypoxia, or oxidative stress, RIPK1-RIPK3-MLKL signalling may become dominant and shift neutrophils toward necroptotic membrane rupture. At the same time, inflammasome activation, gasdermin cleavage, and protease-dependent membrane injury can intersect with this axis, linking the apoptosis-necroptosis-pyroptosis triad to cytokine release, DAMP amplification, and secondary necrosis [137]. Metabolic and redox pressures also bias switching among apoptotic, suicidal NETotic, ferroptotic, and necrotic outcomes; the roles of ROS intensity, ATP availability, mitochondrial dysfunction, and iron-dependent lipid peroxidation are discussed in Section 7.

Epigenetic and chromatin-level regulation may further stabilise these pathological phenotypes, although direct evidence in human bone infection remains limited. DNA methyltransferases (DNMTs), histone modifications, PAD4-dependent chromatin remodelling, and infection-induced transcriptional programmes may influence neutrophil maturation, inflammatory priming, NET competence, cytokine output, and resistance to resolution [81,101,140]. These regulatory networks are especially relevant to emergency granulopoiesis and trained myeloid responses, where upstream marrow programming may generate neutrophils already biased toward persistent inflammatory retention or exaggerated lytic responses [71,101]. Future studies should therefore combine fate markers with chromatin accessibility, DNA methylation, transcriptomic, and spatial profiling to determine whether epigenetic “locking” of neutrophil states contributes to chronic osteomyelitis and implant-associated infection [39].

## 7 Stress-driven programmes: ferroptosis and mitochondrial permeability transition-driven necrosis

Ferroptosis is particularly relevant as a stress-linked framework because infected bone combines hypoxia, acidosis, iron dysregulation, glutathione depletion, and abundant oxidants. Together, these conditions may compromise the GSH–GPX4 antioxidant axis, increase the intracellular labile iron pool, and thereby promote lipid peroxidation [10,45,142]. Even before complete cell death occurs, lipid peroxidation-driven dysfunction may impair NADPH oxidase- and myeloperoxidase-dependent antibacterial activity, helping explain why neutrophils may be present but functionally exhausted in chronic bone infection [10,120].

Mitochondrial permeability transition-driven necrosis represents another stress-linked endpoint. Although mature neutrophils are glycolysis-dominant, their mitochondria remain important signalling hubs [43]. Under severe oxidative stress, calcium overload, and ONOO^−^ accumulation, persistent opening of the mitochondrial permeability transition pore can collapse mitochondrial membrane potential, terminate residual ATP production, and cause necrotic membrane failure [43,45]. In infected bone, this process may rapidly convert metabolically stressed neutrophils into a source of HMGB1, histone-DNA complexes, IL-1α, and other DAMPs, thereby amplifying local inflammatory injury.

More broadly, fate programmes probably interconvert under shared metabolic pressure. ROS excess, iron dysregulation, and ATP depletion can bias stressed neutrophils toward suicidal NETosis or other forms of NET release, or toward irreversible necrotic collapse [46,143]. This is why metabolic context is not just a modifier of neutrophil fate, but a threshold-setting system that gates whether neutrophils remain bactericidal, undergo ordered apoptosis, release NETs, or enter ferroptotic or necrotic collapse. In many lesions it is the dominant variable that decides whether the cell remains useful, becomes destructive, or simply fails [10].

This distinction between neutrophil presence and neutrophil competence is important for interpreting lesion histology and biomarker data, because a neutrophil that survives numerically but enters lipid peroxidation-driven dysfunction may no longer perform the same antibacterial programme as a newly recruited cell [120]. After defining the major neutrophil fate programmes mechanistically, the following clinical sections do not re-describe these pathways in isolation. Instead, they map the same fate programmes onto distinct orthopaedic infection microenvironments to illustrate how clinical presentation, spatial niche, and therapeutic priorities differ across osteomyelitis, biofilm-associated infection, contiguous soft-tissue infection, and fracture-related infection. Because the evidentiary support for several components of this framework remains uneven, Table 2 summarises the available human, animal, and in vitro/ex vivo bone-infection evidence for the most inferentially assigned terminal fates, non-terminal outcomes, and phenotypic states.

**Table 2:** | Evidence grading for selected neutrophil programmes proposed in bone infection.

Neutrophil programme	Classification	Human bone infection	Animal bone infection	In vitro/ex vivo	Overall interpretation
Ferroptosis	Terminal fate	Limited; mainly tissue/transcriptomic associations	Limited preclinical evidence	Mechanistic redox and lipid-peroxidation evidence	Low; neutrophil-specific validation remains limited
MPT-driven necrosis	Terminal fate	Not established	Not established in bone infection	Indirect mechanistic support	Very low; largely extrapolative
Necroptosis	Terminal fate	Limited; mainly pathway-level evidence	Limited infectious/inflammatory evidence	Mechanistic RIPK1/RIPK3/MLKL evidence	Low; direct neutrophil evidence remains sparse
Reverse migration	Non-terminal tissue-exit outcome	Not established	Not established in bone infection; indirect support from non-skeletal models	Limited	Very low; inferential in bone infection
Antigen-presenting neutrophils	Phenotypic/activation state	Limited; direct spatial/functional evidence lacking	Indirect; mainly non-orthopaedic models	Limited functional evidence	Very low–low; largely extrapolated

Evidence categories are descriptive rather than formal quality grades: “direct” indicates evidence obtained within human or experimental bone/orthopaedic infection; “limited” indicates sparse or non-lineage-resolved bone-infection evidence; “indirect” indicates evidence derived primarily from related infectious or inflammatory models; and “not established” indicates that direct bone-infection evidence was not identified among the studies reviewed.

## 8 Osteomyelitis

Osteomyelitis provides the clearest example of why neutrophil fate must be interpreted spatially (Fig. 2). Chronic lesions are not biologically uniform. They contain necrotic bone, poorly perfused inflammatory margins, vascularised reparative regions, and variable bacterial density. A useful conceptual model is therefore to think in terms of partially overlapping microenvironments rather than a single neutrophil response [41,42].

**Fig. 2:**
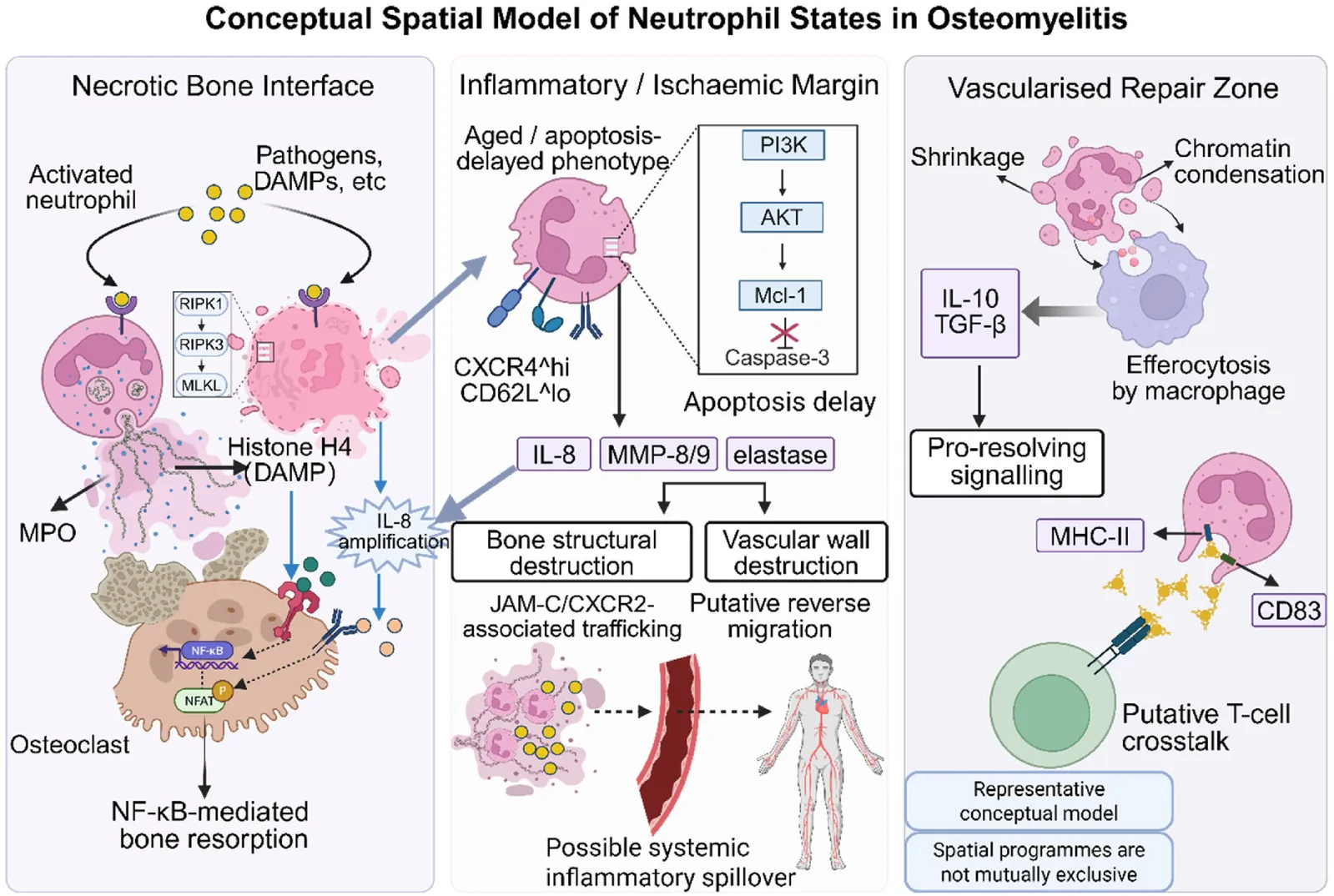
| Conceptual spatial model of osteomyelitis.

Chronic osteomyelitic lesions contain partially overlapping microenvironments, including necrotic bone interfaces, poorly perfused inflammatory margins, and more vascularised peripheral repair zones. NET-associated inflammation and neutrophil-rich osteolytic interfaces are supported by orthopaedic infection studies, whereas the proposed enrichment of necroptosis, apoptosis-delayed or aged phenotypes, and efferocytosis-associated peripheral zones remains partly inferential. The model should therefore be interpreted as a hypothesis-generating spatial framework rather than as a fixed anatomical map or temporal sequence.

Abbreviations: AKT, protein kinase B; BM, bone marrow; CD62L, L-selectin; CD83, cluster of differentiation 83; CXCR2, C-X-C chemokine receptor 2; CXCR4, C-X-C chemokine receptor 4; DAMP, damage-associated molecular pattern; IL, interleukin; JAM-C, junctional adhesion molecule C; Mcl-1, myeloid cell leukaemia 1; MHC-II, major histocompatibility complex class II; MLKL, mixed lineage kinase domain-like protein; MMP, matrix metalloproteinase; MPO, myeloperoxidase; NFAT, nuclear factor of activated T cells; NF-κB, nuclear factor kappa B; PI3K, phosphoinositide 3-kinase; RIPK1/3, receptor-interacting protein kinases 1 and 3; TGF-β, transforming growth factor beta.

### 8.1 Spatial organisation of neutrophil states in osteomyelitic lesions

At the necrotic interface, neutrophils are exposed to high concentrations of microbial products, extracellular DNA, DAMPs, lactate, acidity, and severe oxygen restriction. Neutrophil-rich inflammation and NET-associated tissue injury have been documented in infected skeletal lesions, while the DAMP-rich, hypoxic, and poorly cleared microenvironment may also favour necroptotic and other lytic programmes [37,41].

NET formation at the necrotic interface may provide short-term containment by trapping exposed bacteria and concentrating MPO, elastase, and other antimicrobial factors near the nidus [22,61]. Yet the same products can also damage viable bone margins and provide new inflammatory substrate for osteoclastogenesis [14]. The clinical correlate is a lesion that appears simultaneously purulent and locally destructive. From a therapeutic perspective, this compartment is the least likely to respond to systemic antibiotics or generic immunosuppression alone. It is the compartment that often requires debridement, dead-space management, and local control of both microbes and host-derived matrix injury [8,45,105].

In the inflammatory margin, neutrophils are more likely to encounter survival cytokines and intermediate oxygen levels. Here, delayed apoptosis and aged activated phenotypes may become prominent [129,131,133]. These cells can continue to produce IL-8, elastase, MMPs, and osteoclast-promoting signals, thereby prolonging inflammation beyond the phase in which intense neutrophil activation is most beneficial for pathogen control [114,118]. This zone is therefore a likely source of ongoing bone resorption and lesion expansion [138]. Apoptosis-delayed and aged neutrophils are particularly well positioned to drive osteolysis because they persist close to viable bone and reparative tissue rather than remaining confined to the purulent core [114]. Their continued secretion of S100 proteins, matrix metalloproteinases, elastase, and IL-1 family mediators can amplify monocyte recruitment and bias local myeloid differentiation toward inflammatory osteoclast programmes [114,118,138]. This compartment may therefore be a better target for pro-resolution therapy than the necrotic interface itself, because neutrophils here are still biologically plastic and remain in closer contact with vascular and stromal elements that can be therapeutically redirected.

Closer to vascularised marrow and reparative tissue, neutrophils are more likely to undergo apoptosis and efferocytosis, and in some settings they may acquire non-classical immunoregulatory features [75]. This outer zone may still contain residual infection, but its microenvironment is more favourable to inflammatory resolution than the necrotic core. The biological balance between these compartments probably determines whether a lesion stabilises, reforms bone, or enters a chronic destructive state.

Osteomyelitis is particularly suited to a fate-based framework because of its pronounced spatial compartmentalisation. Necrotic bone, suppurative exudate, granulation tissue, vascularised marrow, and remodelling cortex can coexist within the same lesion. The biological question is therefore not simply whether neutrophils are present, but which neutrophil programmes predominate in each compartment and how these compartments interact over time [97]. Integrating oxygenation, pH, vascular access, matrix composition, and bacterial architecture with neutrophil state may provide a more informative view of lesion heterogeneity than averaging the entire lesion into a single inflammatory readout [[49], [50], [51]].

### 8.2 Temporal transitions from acute containment to chronic injury

Time is as important as location. Early in infection, rapid recruitment, phagocytosis, degranulation, and limited NET formation may still be adaptive [22,60,61]. With persistent bacterial burden, however, hypoxia deepens, macrophage clearance fails, and pro-survival signals accumulate [42,131]. The lesion then shifts from a containment programme toward a self-sustaining inflammatory loop. In that phase, the same neutrophil functions that were initially protective can become a major source of host damage.

Spatial lesion zoning should be viewed as complementary to, rather than superior to, temporal staging. Temporal staging remains clinically useful for distinguishing early contamination, acute containment, chronic persistence, and repair failure. However, chronic bone infection frequently contains asynchronous compartments within the same lesion, including purulent exudate, necrotic bone, sequestrum-adjacent interfaces, biofilm-associated implant surfaces, ischaemic inflammatory margins, vascularised marrow, and reparative callus [8,45,88,105]. A single temporal label may therefore obscure biologically distinct microenvironments that coexist within one lesion. Spatial zoning is proposed here as an interpretive framework for intralesional heterogeneity rather than as a validated clinical staging system. Whether it provides superior predictive performance over temporal staging alone remains unproven and requires prospective validation.

Diagnostic imaging may help localise lesion compartments but cannot directly assign neutrophil fate. MRI findings such as bone marrow oedema, abscess formation, sinus tracts, cortical destruction, sequestrum, involucrum, and soft-tissue enhancement may indicate inflammatory burden, tissue necrosis, vascular compromise, and anatomical compartmentalisation. PET-CT, leukocyte-based nuclear imaging, and related metabolic imaging approaches may reveal spatial gradients of inflammatory activity, but these signals remain indirect correlates rather than specific readouts of NETosis, apoptosis, necroptosis, ferroptosis, or efferocytosis. Investigational fluorescence-guided, hypoxia-sensitive, protease-responsive, matrix-targeted, or myeloid-cell-associated probes may eventually guide targeted intraoperative sampling of distinct lesion compartments, although their utility in orthopaedic infection remains to be validated.

Early lesions may still be amenable to fate-directed intervention before the pathological microenvironment becomes structurally entrenched. Long-standing lesions progressively become structurally encoded, with necrotic bone and fibrotic boundaries locking the tissue into a chronic destructive state [8,21,88,105].

## 9 Biofilm-associated bone and implant infection

Implant-associated infection adds another layer of complexity because neutrophils encounter not only bacteria but also a structured extracellular matrix that impairs phagocytosis and traps immune effectors [9,21,90,103,104,[107], [108], [109]]. The biofilm matrix sequesters opsonins, limits penetration of antimicrobial molecules, alters local pH and oxygen gradients, and promotes frustrated phagocytosis [90,102,108,109]. Persistent host–microbe–material inflammatory signalling can sustain neutrophil recruitment and local retention, whereas the biofilm matrix impairs phagocytosis and antimicrobial function, leading to the accumulation of abundant but ineffective neutrophils [103].

### 9.1 Surface, intermediate, and deep biofilm-associated neutrophil states

The surface–intermediate–deep organisation described below provides a conceptual framework for interpreting neutrophil behaviour around mature biofilms. Direct orthopaedic infection studies support the general principles that biofilms impair phagocytosis, trap or remodel neutrophil-derived material, and allow abundant but ineffective neutrophil accumulation at infected implant interfaces, whereas specific depth–fate assignments remain inferential [[102], [103], [104],[107], [108], [109]].

At the biofilm surface, neutrophils remain in closest contact with host tissue and newly adherent bacteria. They may retain partial oxidative and degranulation capacity, but phagocytic efficiency is already impaired. This state can be viewed as partially activated but functionally constrained [108,109].

Within intermediate biofilm layers, NET-associated material may accumulate and become incorporated into the extracellular matrix (Fig. 3) [21,104,106,107]. The mechanisms by which NETs shift from containment to biofilm reinforcement are discussed in Section 9.2.

**Fig. 3:**
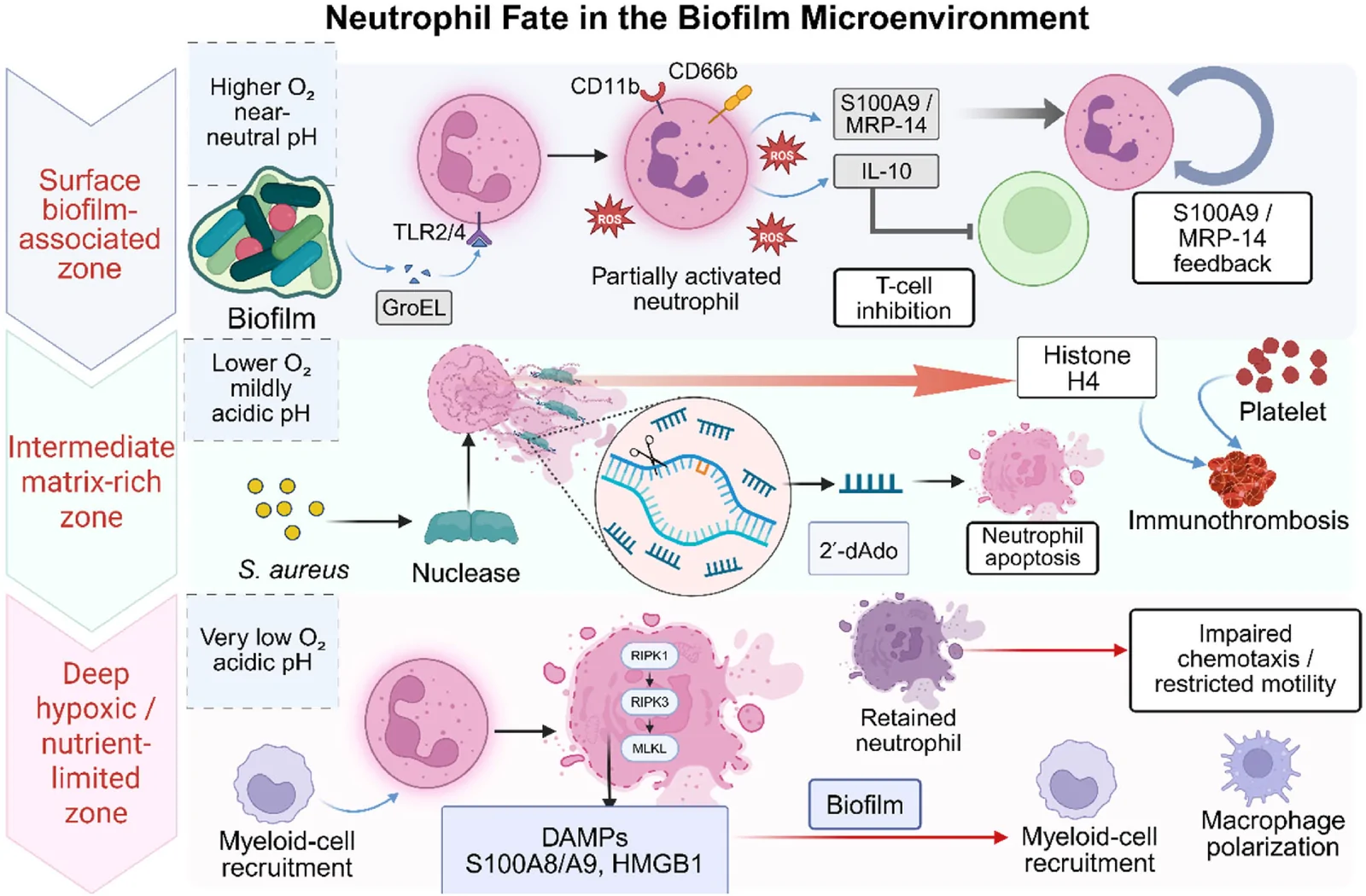
Qualitative model of neutrophil states across a mature biofilm microenvironment.

In deeper biofilm-associated compartments, extreme hypoxia, nutrient limitation, and chemotactic failure likely favour inflammatory retention and metabolic exhaustion that may predispose neutrophils to necroptotic or necrotic collapse [31,77,101,102]. The resulting DAMP-rich environment can recruit suppressive myeloid populations and further exclude effective clearance [77]. This is one mechanism by which chronic implant infection acquires both immune persistence and antibiotic tolerance [21,106].

Mature biofilm-associated infection creates spatial heterogeneity in bacterial accessibility, oxygenation, pH, nutrient availability, extracellular DNA, extracellular polymeric matrix, and host-derived inflammatory mediators. Surface biofilm-associated regions are relatively more oxygenated and may permit partial neutrophil activation, although effective phagocytosis remains restricted. Intermediate matrix-rich regions may favour NET accumulation and incorporation of NET-associated material into the extracellular matrix, whereas deep hypoxic and nutrient-limited regions may promote impaired chemotaxis, restricted motility, inflammatory retention, metabolic stress, and necroinflammatory collapse. Neutrophil-derived alarmins, including S100A8/A9 and HMGB1, may reinforce inflammatory feedback and myeloid-cell recruitment. Depth-specific fate assignments remain conceptual and are extrapolated from biofilm, in vitro, and animal studies rather than direct spatial mapping in human implant-associated infection.

**Abbreviations:** 2′-dAdo, 2′-deoxyadenosine; CD11b, cluster of differentiation 11b; CD66b, cluster of differentiation 66b; DAMPs, damage-associated molecular patterns; GroEL, bacterial 60-kDa chaperonin; HMGB1, high-mobility group box 1; IL, interleukin; MLKL, mixed lineage kinase domain-like protein; MRP-14, migration inhibitory factor-related protein 14; RIPK1/3, receptor-interacting protein kinases 1 and 3; ROS, reactive oxygen species; S100A8/A9, S100 calcium-binding proteins A8 and A9; TLR2/4, Toll-like receptors 2 and 4.

### 9.2 When NETs shift from containment to biofilm reinforcement

The dual role of NETs is central to biofilm infection. During the earliest phase, NETs can immobilise surface bacteria and concentrate antimicrobial proteins [26,90,100,[104], [105], [106], [107],^144^]. With ongoing infection, bacterial nucleases, extracellular DNA recycling, platelet activation, and immunothrombotic remodelling can convert this response into a pro-biofilm state [108]. Once that transition occurs, increasing neutrophil numbers alone is unlikely to restore effective antimicrobial control. The more relevant goal is to interrupt the feedback loop between NET release, NET degradation, and biofilm reinforcement [87]. Neutrophils should therefore be viewed as active participants in a maladaptive host–microbe–material architecture rather than as passive victims of biofilm.

Foreign-body reaction intensifies this problem. Even in the absence of heavy planktonic burden, the implant interface can maintain low-grade inflammatory signalling through adsorbed host proteins, wear particles, surface roughness, and micromotion. These cues alter neutrophil adhesion, promote frustrated phagocytosis, and modify crosstalk with macrophages and foreign-body giant cells [18,19,42]. Biofilm formation can then further reinforce inflammation at this already activated interface. Once this interface becomes structurally organised around persistent neutrophil activation and matrix protection, it functions as both a microbial and an immune–material niche; the implications for implant retention or removal are discussed in Section 12.1.

Implant-associated infection also highlights the importance of the host-microbe-material triad. Surface topography, corrosion products, wear debris, and local micromotion can all influence how neutrophils behave at the interface. Some bacteria are also able to persist intracellularly within synoviocytes, osteoblasts, or macrophages adjacent to biofilm. This creates a relay system in which extracellular biofilm and intracellular reservoirs perpetuate one another [5,6].

Thus, abundant NET material at a chronic implant interface should be interpreted as evidence of maladaptive host–microbe–material interactions rather than effective bacterial clearance [105,144].

## 10 Soft-tissue infection contiguous with bone

For soft-tissue infection contiguous with bone, clinical studies of diabetic foot infection and chronic wounds support hypoxia, ischaemia, impaired bacterial clearance, excessive protease activity, NET accumulation, and defective resolution in poorly healing wounds [52,[110], [111], [112],^132^]. Precise zone-specific neutrophil-state and fate-related assignments, however, remain inferential and are derived mainly from chronic wound biology, diabetic inflammation, and experimental infection models [52,[110], [111], [112]].

Soft-tissue infection frequently precedes or accompanies osteomyelitis, particularly in diabetic foot disease, pressure injury, and traumatic wounds (Fig. 4) [111,112]. Its distinctive feature is the convergence of hyperglycaemia, advanced glycation products, vascular insufficiency, recurrent tissue ischaemia, and impaired macrophage clearance. These conditions impair chemotaxis and phagocytosis while biasing neutrophils towards delayed-clearance and NET-rich states, thereby compromising bacterial clearance, sustaining tissue injury, and facilitating extension of infection toward bone [52,111,112,145,146].

**Fig. 4:**
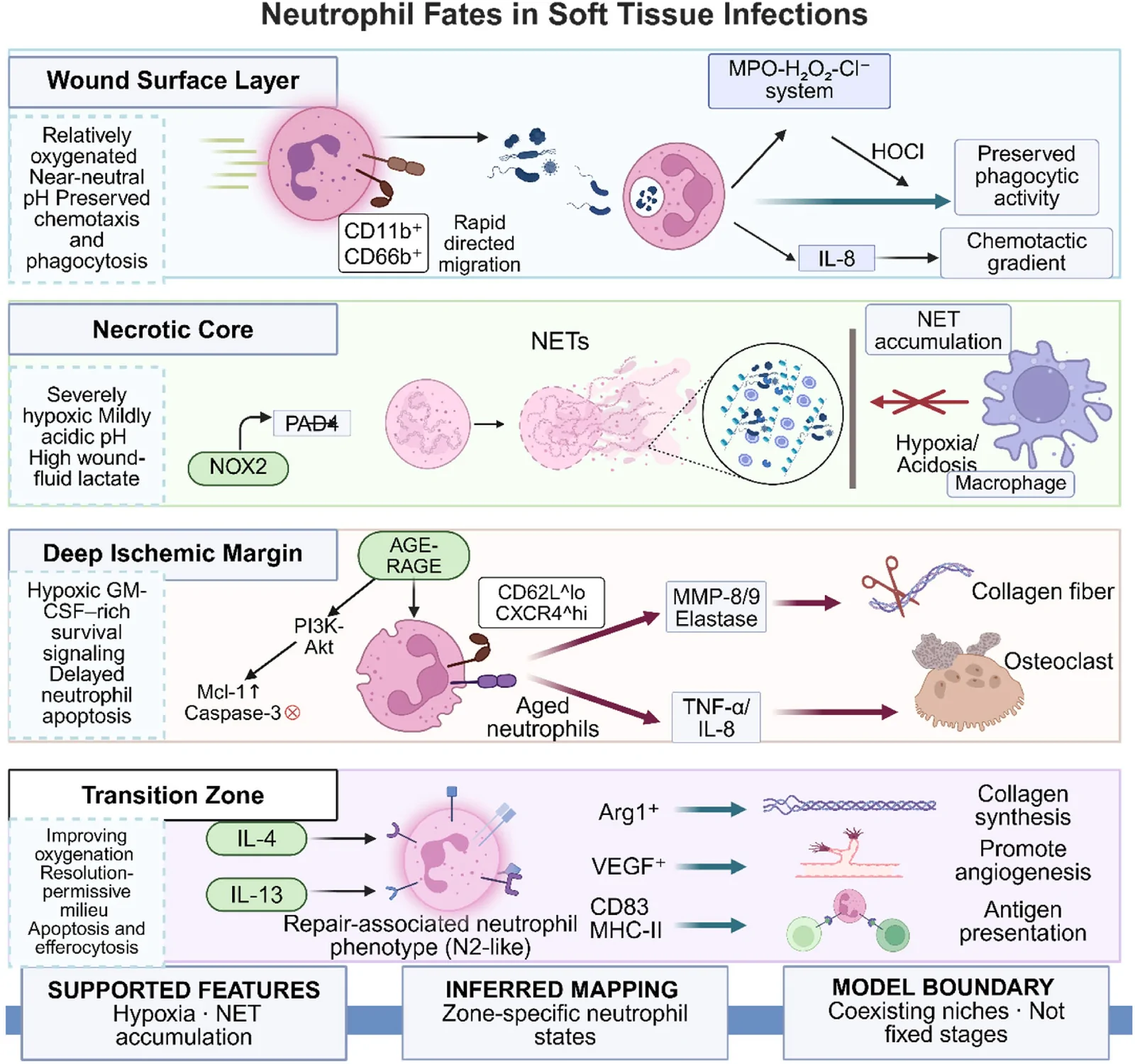
Spatial organisation of neutrophil states and fate-related outcomes in soft-tissue infection contiguous with bone.

The schematic depicts four partially overlapping niches: wound-surface, necrotic-core, ischaemic-margin, and bone-facing transition zones, each associated with representative neutrophil functions and fate-related states. These zones represent coexisting spatial niches rather than fixed chronological stages. The lower band distinguishes supported lesion features, including hypoxia and NET accumulation, from inferred zone-specific neutrophil-state assignments. The model is therefore intended as a hypothesis-generating spatial synthesis rather than a definitive spatial or temporal atlas of human disease.

Abbreviations: AGE–RAGE, advanced glycation end product–receptor for advanced glycation end products; Arg1, arginase 1; CD11b, cluster of differentiation 11b; CD62L, L-selectin; CD66b, cluster of differentiation 66b; CD83, cluster of differentiation 83; CXCR4, C-X-C chemokine receptor 4; GM-CSF, granulocyte–macrophage colony-stimulating factor; HOCl, hypochlorous acid; IL, interleukin; Mcl-1, myeloid cell leukaemia 1; MHC-II, major histocompatibility complex class II; MMP, matrix metalloproteinase; MPO, myeloperoxidase; NETs, neutrophil extracellular traps; NOX2, NADPH oxidase 2; PAD4, protein arginine deiminase 4; PI3K–Akt, phosphoinositide 3-kinase–protein kinase B signalling; TNF-α, tumour necrosis factor alpha; VEGF, vascular endothelial growth factor.

## 11 Fracture-related infection

Fracture-related infection sits at the intersection of trauma biology and infection biology. Neutrophils enter a tissue bed that has already been altered by haemorrhage, devascularisation, necrosis, implant fixation, and local release of alarmins. Fracture-related infection differs from established osteomyelitis because neutrophils enter a tissue that is simultaneously contaminated and trying to heal. In the first hours after trauma, they are required to clear inoculated bacteria, remove damaged tissue, and coordinate early inflammatory recruitment. The fracture haematoma is not simply debris. It is a provisional regenerative niche whose composition influences angiogenesis and callus formation [95]. Early neutrophil recruitment is essential for bacterial containment, but excessive or persistent activation can also compromise fracture healing by intensifying proteolysis, oxidative injury, and osteoclastogenic signalling [95].

Compared with established osteomyelitis, the defining feature of fracture-related infection is not only biofilm but also unstable tissue repair. Neutrophil fate therefore needs to be interpreted against the requirements of bone regeneration. A response that might be acceptable in a chronically infected implant interface may be highly detrimental in a fresh fracture where callus formation, angiogenesis, and mechanical stability are still developing [92,93,147,148].

As fracture-related infection evolves, the same determinants seen in osteomyelitis and implant infection come into play, but in a biomechanically unstable environment. Hardware choice and construct stability probably also influence neutrophil fate more than is usually appreciated. Stable fixation can reduce repeated tissue injury, dead space, and DAMP release. Unstable constructs or repeated revisions do the opposite and may continually reset the lesion toward fresh neutrophil recruitment without permitting resolution. Motion, dead space, ischaemia, and biofilm on fixation devices all favour prolonged neutrophil recruitment [16]. These cells then act not only on bacteria but also on callus, periosteum, and nascent vasculature. Persistent NET accumulation and protease release can degrade provisional matrix, interfere with osteoblast function, and maintain a cytokine milieu incompatible with bridging union [94,95]. This helps explain why fracture-related infection often presents clinically as both infection control failure and repair failure. Infection-related nonunion may therefore reflect not only mechanical or microbiological failure, but also a failure to re-establish coordinated neutrophil–macrophage responses that support callus maturation and vascularised bone repair.

## 12 Therapeutic opportunities

For the therapeutic strategies discussed below, we separately grade the evidence base and translational maturity. Human or clinical evidence refers to findings from patient tissues, clinical orthopaedic infection samples, or established clinical practice relevant to infection control and tissue reconstruction. Animal evidence refers to in vivo osteomyelitis, implant-associated infection, fracture-related infection, or wound-infection models. In vitro evidence refers to neutrophil, macrophage, biofilm, biomaterial, or co-culture systems. Conceptual strategies are those derived mainly from related inflammatory, infectious, or immunometabolic models and still requiring direct validation in bone infection. Table 3 therefore reports both the main evidence base and the current translational/developmental stage for each strategy, rather than presenting all approaches as equally mature.

**Table 3:** | Evidence grading and translational maturity of therapeutic strategies targeting neutrophil fate in bone and implant infection.

Strategy class	Intended neutrophil effect	Main evidence base	Current translational/developmental stage	Most relevant orthopaedic setting	Key limitation
NET-targeted intervention and NET clearance	Reduce persistent NET formation or accumulation after early containment while preserving antimicrobial defence	Human tissue and clinical association studies support NET accumulation in orthopaedic infection and chronic wounds; therapeutic targeting is mainly supported by in vitro and animal studies	In vitro and rodent models; limited human tissue association data; no established orthopaedic clinical trial evidence	Biofilm-associated infection, chronic wound-contiguous infection, implant-associated osteolysis	Premature NET suppression may impair early bacterial containment; timing and species-specific NET regulation remain unresolved
Apoptosis restoration and efferocytic clearance	Restore timely apoptosis and macrophage-mediated removal of aged or apoptosis-delayed neutrophils	Strong mechanistic evidence from inflammation-resolution biology; limited direct therapeutic evidence in bone infection	Mechanistic and in vitro studies; early rodent inflammatory or infection models; limited orthopaedic-specific validation	Chronic osteomyelitis, inflammatory lesion margins, persistent postoperative inflammation	Requires biomarkers that distinguish protective recruitment from maladaptive persistence
Pro-resolution tissue-repair modulation	Promote macrophage-mediated resolution, angiogenesis, stromal recovery, and bone repair after source control	Broader inflammation-resolution and wound-repair evidence; limited direct orthopaedic infection validation	Mechanistic, in vitro, and early animal studies; limited human bone-infection evidence	Post-debridement repair zones, fracture-related infection, infection-associated nonunion	Premature repair signalling may be harmful when bacterial burden or necrotic tissue persists
Metabolic or redox modulation	Limit stress-driven inflammatory collapse while preserving controlled antimicrobial function	Mainly in vitro and animal evidence from hypoxia, ROS, metabolic, wound, and nanomaterial models	In vitro systems and rodent wound or bone-infection models; selected responsive biomaterials in preclinical animal testing	Hypoxic bone lesions, diabetic or ischaemic wounds, devascularised tissue	Local delivery, off-target effects, and species-specific metabolic thresholds require human validation
Necroptosis modulation	Reduce RIPK1/RIPK3/MLKL-mediated membrane rupture and DAMP amplification	Predominantly mechanistic, in vitro, and rodent inflammatory or infectious evidence; sparse direct human bone-infection evidence	Preclinical; no established orthopaedic clinical evidence	Necroinflammatory bone interfaces and severe tissue-destructive infection	Pathway dominance in human neutrophils and effects on microbial control remain uncertain
Trafficking and inflammatory-retention modulation	Reduce pathological lesion retention or restore controlled tissue disposition without impairing recruitment	Mainly non-skeletal inflammatory models; limited direct bone-infection evidence	Conceptual to early preclinical and hypothesis-generating	Vascularised inflammatory margins rather than necrotic or mature biofilm compartments	Surface markers cannot establish tissue exit; impaired containment or systemic dissemination are potential risks
Host-defence priming or trained immunity	Improve antimicrobial readiness without prolonged tissue-damaging activation	Mechanistic immune-training studies and non-orthopaedic animal models; limited orthopaedic-specific evidence	Conceptual to early preclinical; no established orthopaedic large-animal or clinical trial evidence	High-risk infection prevention, revision surgery, recurrent implant infection	Specificity, durability, reversibility, and risk of inflammatory amplification remain insufficiently defined
Biomaterial-assisted delivery and anti-biofilm platforms	Improve local delivery, disrupt lesion barriers, facilitate target exposure or cellular uptake, and support osseointegration	Strong in vitro biofilm and biomaterial evidence with increasing rodent implant-infection evidence	In vitro and rodent models; selected platforms approaching large-animal evaluation; limited clinical evidence	Periprosthetic joint infection, implant-associated osteomyelitis, dead-space management, revision surgery	Requires source control; penetration, release kinetics, biocompatibility, osseointegration, and human neutrophil responses require validation

Against this evidence hierarchy, translation of neutrophil-directed therapy requires a delivery-aware framework. The central challenge is not only selecting an immunomodulatory target, but also reaching the lesion compartment in which the maladaptive neutrophil programme is active. Necrotic and poorly perfused bone, dense extracellular matrix, fibrin, extracellular DNA, mature biofilm, acidic and hypoxic tissue, and poorly drained dead space can restrict systemic drug access and alter payload stability or activity. Effective intervention should therefore follow an integrated sequence: identification of the dominant lesion compartment and disease stage; surgical source control and reduction of physical and biological barriers; local or biomaterial-assisted delivery when systemic exposure is inadequate; engagement of the intended extracellular or intracellular neutrophil target; and validation of antimicrobial control, inflammatory resolution, osseointegration, and bone repair [96,97,149].

To avoid treating mechanistically distinct interventions as equivalent, this therapeutic framework distinguishes three complementary roles. Direct fate- or pathway-modulating strategies include NET-targeted intervention and NET clearance, apoptosis restoration and efferocytic clearance, pro-resolution tissue-repair modulation, metabolic or redox modulation, necroptosis modulation, and trafficking or inflammatory-retention modulation. Host-defence priming or trained immunity is considered an upstream host-conditioning strategy rather than an acute fate-targeting intervention [150]. Biomaterial-assisted delivery and anti-biofilm platforms are considered delivery-enabling and lesion-modifying strategies that facilitate target exposure, cellular uptake where required, and pathway engagement after adequate source control. Table 3 grades all strategy categories according to their evidence base and translational or developmental stage. Fig. 5 instead visualises the major direct fate- or pathway-modulating modules within a delivery-aware sequence of lesion access, local or biomaterial-assisted delivery, matrix or biofilm penetration, neutrophil exposure or uptake, and pathway engagement. In this figure, biomaterial-assisted delivery and anti-biofilm platforms are represented as the delivery-enabling layer, whereas host-defence priming or trained immunity remains an upstream host-conditioning strategy graded in Table 3 but is not depicted as an acute fate-modulating module [96,97,141,149].

**Fig. 5:**
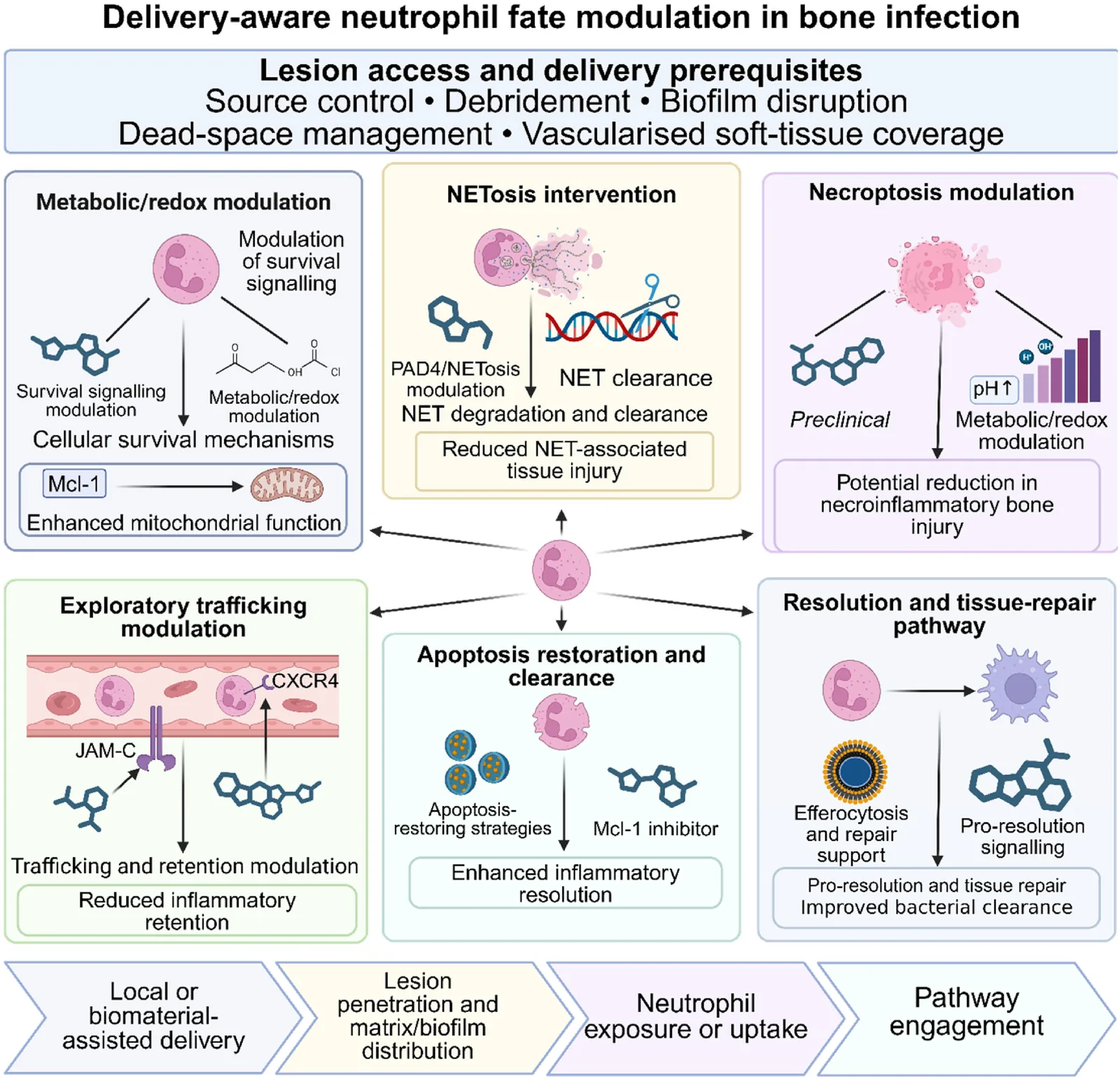
| Delivery-aware framework for neutrophil fate modulation in bone infection.

### 12.1 From neutrophil fate to orthopaedic decision-making

The translational value of a neutrophil fate framework lies in its ability to reinterpret established orthopaedic infection principles through the biology of the local tissue microenvironment. Debridement, for example, should be viewed not only as a method for reducing bacterial burden, but also as a way to remove necrotic bone, fibrin, extracellular DNA, biofilm matrix, and DAMP-rich tissue compartments that select for persistent NET formation and accumulation, delayed apoptosis, and other lytic inflammatory programmes. In this sense, surgical source control may remodel the local microenvironment by removing factors that favour maladaptive neutrophil states and lytic inflammatory programmes, thereby creating conditions more favourable to antimicrobial efficacy, efferocytic clearance of apoptotic neutrophils, angiogenesis, and bone repair [149].

This concept is particularly relevant to implant-associated infection and periprosthetic joint infection. In early infection, when the implant is stable and the biofilm is still immature, implant retention strategies combined with debridement, exchange of modular components, and local antimicrobial delivery may preserve a microenvironment in which neutrophils retain useful phagocytic and antimicrobial functions. By contrast, in chronic infection with mature biofilm, implant loosening, necrotic interfaces, recurrent sinus formation, or failed osseointegration, the implant surface may function as a persistent host-microbe-material scaffold for frustrated neutrophil activation, NET accumulation, and ineffective inflammatory retention [8,88,100,105]. In such settings, implant removal or staged implant exchange may disrupt the pathological host–microbe–material niche that sustains maladaptive neutrophil activation and inflammatory persistence.

Dead-space management, local antibiotic delivery, and soft-tissue coverage can also be integrated into this framework. Dead space favours hypoxia, fluid accumulation, poor immune-cell clearance, and persistence of necrotic debris, all of which may prolong neutrophil survival or divert neutrophils toward NET-rich and necroinflammatory states. Local antibiotic carriers, spacers, and dead-space fillers therefore do more than increase antimicrobial concentration. They may also reshape the tissue chemistry that determines whether infiltrating neutrophils remain bactericidal, become trapped in destructive inflammation, or transition toward resolution [144,[151], [152], [153]]. Similarly, vascularised soft-tissue coverage improves oxygen delivery, antibiotic penetration, leukocyte trafficking, and macrophage-mediated clearance of apoptotic neutrophils, whereas ischaemic or poorly covered wounds sustain hypoxia, acidosis, protease accumulation, NET retention, and defective efferocytosis [52,109,110]. For fracture-related infection and infection-associated nonunion, stable fixation and restoration of a repair-permissive immune niche remain essential [92,93,147,148]; the disease-specific interaction between persistent neutrophil activation and callus failure is discussed in Section 11. Thus, neutrophil fate should be considered a practical design variable in orthopaedic infection management. It provides a biological link between source control, implant strategy, dead-space management, local antibiotic delivery, soft-tissue reconstruction, and prevention of infection-associated nonunion [37,147].

### 12.2 Mouse-to-human translational barriers in neutrophil-targeted therapy

A major translational barrier is that many mechanistic studies of neutrophil lytic death, including pyroptosis, necroptosis, ferroptosis, and suicidal NETosis, rely heavily on rodent models. These models are indispensable for defining pathway logic, but murine neutrophils should not be considered interchangeable with human neutrophils. Neutrophils account for approximately 50–70% of circulating leukocytes in humans but only about 10–25% in mice, which can alter systemic inflammatory tone, recruitment kinetics, and the apparent therapeutic window of neutrophil-targeted interventions. Human and murine neutrophils also differ in granule composition, defensin expression, scavenger receptors, Fc receptors, chemokine receptor usage, and the thresholds by which bacterial products, DAMPs, hypoxia, oxidants, and biomaterial surfaces trigger lytic or non-lytic programmes [58,60,63,64,79].

These differences are particularly important when designing targeted immunomodulatory biomaterials. A material that suppresses NET formation, blocks inflammasome signalling, modulates redox stress, or redirects macrophage-neutrophil crosstalk in mice may not produce the same balance between bacterial control and tissue protection in human orthopaedic infection. Therefore, rodent data should be used to generate mechanistic hypotheses rather than to define human therapeutic dosing, timing, or safety [147,148]. Future translational studies should incorporate human neutrophils, patient-derived synovial fluid or bone-infection samples, ex vivo biofilm-biomaterial systems, and cross-species validation of key pathway readouts before advancing neutrophil-targeted immunomodulatory materials toward clinical use [141].

### 12.3 NET-targeted intervention and NET clearance

NET-directed approaches include inhibition of excessive NET formation, degradation or clearance of persistent extracellular DNA and NET-associated material, and reduction of NET-promoting redox stress [140,149,154]. These interventions are most relevant after the phase of useful pathogen containment has passed, particularly in biofilm-rich infection and chronic wounds in which persistent NET material contributes to matrix accumulation and tissue injury [139]. Their principal limitation is timing: premature NET suppression may weaken early bacterial containment, whereas delayed intervention may no longer be sufficient to reverse established biofilm architecture and structural tissue damage [96,140]. NET-targeted intervention therefore has a comparatively strong disease-level rationale in orthopaedic infection, although direct therapeutic evidence in human bone infection remains limited [37].

### 12.4 Apoptosis restoration and pro-resolution tissue repair

One therapeutic objective is to restore physiological neutrophil turnover. Strategies that reduce inappropriate survival signalling, including excessive Mcl-1-dependent survival, restore apoptosis, or enhance macrophage-mediated efferocytosis may help remove aged or apoptosis-delayed neutrophils that perpetuate osteolytic inflammation [122,131]. This approach seeks to terminate persistent inflammation through controlled neutrophil clearance rather than broad immunosuppression [57,96,155]. Its rationale is supported by the wider inflammation-resolution literature, but direct therapeutic validation in human bone infection remains limited.

Pro-resolution tissue-repair modulation is related to, but distinct from, apoptosis restoration. Whereas apoptosis-restoring strategies act primarily on neutrophil survival and clearance, pro-resolution interventions aim to support the downstream macrophage, endothelial, stromal, and osteogenic programmes required for inflammatory resolution, angiogenesis, and bone repair after bacterial burden and necrotic tissue have been adequately controlled. These two modules are therefore shown separately in Fig. 5 and graded separately in Table 3. Pro-resolution therapy should not be initiated as a substitute for source control, because premature activation of repair programmes in a persistently infected lesion could permit microbial persistence [27,28,57,82,91].

Neutrophil-directed strategies are positioned as adjuncts to surgical source control, debridement, biofilm disruption, dead-space management, and vascularised soft-tissue coverage. Following adequate lesion preparation, local or biomaterial-assisted delivery may facilitate pathway-specific modulation of metabolic/redox stress, NET formation and clearance, necroptosis, trafficking or inflammatory retention, apoptosis and clearance, and resolution-associated repair. The corresponding therapeutic strategies and their evidence base and translational maturity are summarised in Table 3; the illustrated effects represent therapeutic objectives rather than established clinical efficacy.

Abbreviations: CXCR4, C-X-C chemokine receptor 4; JAM-C, junctional adhesion molecule C; Mcl-1, myeloid cell leukaemia 1; NETs, neutrophil extracellular traps; PAD4, protein arginine deiminase 4.

### 12.5 Metabolic and redox modulation

Because neutrophil fate is closely linked to microenvironmental stress, metabolic and redox modulation represents a distinct therapeutic category. Redox-active nanomaterials, oxygen- or reactive oxygen species-responsive systems, and local microenvironmental repair strategies may alter the balance between controlled antimicrobial function and stress-driven inflammatory collapse [141,151,152,155]. Whereas strategies aimed at restoring timely neutrophil apoptosis act more directly on survival and apoptotic signalling, metabolic and redox interventions seek to modify upstream microenvironmental and cellular stresses, including hypoxia, oxidative imbalance, mitochondrial dysfunction, lipid peroxidation, and metabolic exhaustion [141,[151], [152], [153],^156^]. At present, metabolic and redox modulation should be considered an early experimental strategy for bone infection, because most evidence derives from in vitro systems, animal models, wound models, and biomaterial-based studies rather than human orthopaedic infection trials [151,152,155].

### 12.6 Additional preclinical and exploratory strategies

Necroptosis modulation aims to reduce RIPK1/RIPK3/MLKL-mediated membrane rupture and DAMP release, thereby limiting downstream necroinflammatory amplification [35,38,137,140]. Although necroptotic signalling is mechanistically relevant to inflammatory tissue injury, direct evidence that neutrophil necroptosis is a dominant and therapeutically actionable driver in human osteomyelitis or implant-associated infection remains sparse. Necroptosis-directed intervention should therefore be classified as preclinical rather than presented alongside more clinically grounded infection-management strategies.

Trafficking and inflammatory-retention modulation seeks to reduce pathological neutrophil persistence within vascularised inflammatory margins or to restore controlled tissue disposition without impairing recruitment to active infection. Potential targets include JAM-C- and chemokine-receptor-associated trafficking pathways [44,98,119,135]. However, altered CXCR2, CXCR4, ICAM-1, JAM-C, or CD62L expression cannot by itself establish reverse migration or therapeutic tissue exit. Because the rationale is derived mainly from non-skeletal inflammatory models and direct therapeutic evidence in bone infection is limited, this strategy remains exploratory and hypothesis-generating.

Host-defence priming or trained immunity occupies a different conceptual position from direct modulation of a neutrophil death pathway. Its purpose is to condition innate antimicrobial responsiveness before infection, between recurrent episodes, or in selected high-risk settings without inducing prolonged tissue-damaging activation. Evidence in orthopaedic infection remains largely mechanistic or extrapolated from non-orthopaedic models [85]. Questions concerning cellular specificity, duration, reversibility, pathogen dependence, and the risk of amplifying osteolytic inflammation must be resolved before trained-immunity-based approaches can be advanced beyond the conceptual or early preclinical stage.

### 12.7 Biomaterial-assisted delivery and anti-biofilm platforms

Within this delivery-enabling and lesion-modifying layer, injectable hydrogels, degradable local carriers, calcium phosphate or bioactive glass scaffolds, nanoparticle-based systems, immune-responsive implant coatings, and matrix-penetrating formulations may improve local retention, barrier disruption, spatial distribution, and controlled payload release within poorly perfused bone [95,111,[151], [152], [153],[157], [158], [159]]. Responsive systems triggered by enzymes, acidic pH, hypoxia, or reactive oxygen species may further concentrate treatment within infection-associated microenvironments while limiting systemic immunosuppression [35,92,151,152].

Depending on the intended mechanism, these platforms may act extracellularly by disrupting biofilm or NET-rich matrix, or may facilitate neutrophil exposure and uptake to regulate PAD4-dependent NET formation, inflammasome–gasdermin signalling, RIPK1/RIPK3/MLKL-mediated necroptosis, metabolic or redox stress, apoptosis delay, or efferocytosis-compatible clearance [37,39,92,94,111,114,[151], [152], [153], [154], [155], [156],^160^]. The lesion-specific physical and biological barriers described above may nevertheless alter carrier diffusion, degradation, cellular uptake, and payload activity.

Delivery performance and biological engagement should therefore be evaluated together. Relevant readouts include lesion penetration, retention and distribution within matrix or biofilm, biofilm disruption, extracellular target exposure or neutrophil uptake as appropriate, intracellular pathway engagement, bacterial control, preservation of early antimicrobial function, inflammatory resolution, efferocytosis, osseointegration, bone repair, recurrence, and local and systemic safety [161]. Most neutrophil-directed delivery systems remain at the in vitro or early preclinical stage and require validation in spatially defined bone- and implant-infection models using human-relevant neutrophil systems [111,147,148,[151], [152], [153],^156^].

### 12.8 Clinical implementation: patient selection, timing, and response monitoring

Clinical implementation will depend on matching the intervention to lesion stage, anatomical compartment, implant status, microbial architecture, and host-tissue quality. A fate-directed strategy that is appropriate for an early, accessible lesion may be ineffective or harmful in an established biofilm-rich or ischaemic compartment; patient selection and timing are therefore essential [12,34,37,141,162].

Response assessment should extend beyond culture negativity, wound closure, and conventional serum inflammatory markers to determine whether the pathogenic neutrophil programme has resolved [34,162]. Candidate measures include NET burden within the lesion, S100A8/A9-rich exudate, synovial or wound-fluid proteomic signatures, markers of delayed apoptosis, spatially defined neutrophil–macrophage or neutrophil–stromal niches, and imaging correlates of hypoxia or matrix density [12,24,141]. These candidates remain investigational and require prospective validation before they can guide stage-specific treatment.

## 13 Conclusions

Neutrophil fate provides a practical framework for understanding why bone infection can persist despite antimicrobial therapy, surgical source control, and abundant inflammatory-cell recruitment. The key question is not whether neutrophils are beneficial or harmful, but which components of the neutrophil fate framework dominate in a specific lesion compartment and disease stage.

Three key priorities emerge for future research. First, human bone-infection studies should generate spatially resolved neutrophil atlases that integrate histopathology, microbiology, implant status, diagnostic imaging, and biopsy location. Purulent exudate, sequestrum-adjacent tissue, implant membranes, ischaemic wound margins, and healing callus arise from distinct lesion microenvironments and should therefore be analysed separately. Second, the field needs standardised, measurable definitions and readouts for terminal fates and non-terminal tissue-disposition states and outcomes, including apoptosis with subsequent efferocytic clearance, suicidal NETosis, pyroptosis, necroptosis, ferroptosis, reverse migration, and inflammatory retention, while distinguishing non-lytic NET release as an effector process rather than a terminal fate. Third, therapeutic studies should move from pathway nomination to delivery-aware intervention by demonstrating lesion access, target engagement, preservation of antimicrobial control, and compatibility with tissue repair.

Thus, the next translational step is not broad neutrophil suppression, but stage-aware and compartment-specific redirection of maladaptive neutrophil persistence while preserving early antimicrobial defence.

## AI use disclosure

We hereby declare that no generative AI tools were used for core research content, data generation, or image creation during the preparation of this manuscript. Generative AI tools were used solely for linguistic polishing and grammatical revisions, which did not constitute substantive generation of the research content. All work is original, properly authorized where applicable, and in accordance with the journal's academic standards and ethical requirements.

## Funding statement

This work was supported by the 10.13039/501100001809National Natural Science Foundation of China (82272512, 82472463, and U25C2009); the Anhui Provincial Key Research and Development Programme–Clinical Medical Research Translation Specialization (202304295107020020); the 10.13039/100005622Anhui Provincial Health Research Programme (AHWJ2024Aa10144); the Anhui Provincial Scientific Research Compilation Project (2022AH010076); the USTC Research Funds of the Double First-Class Initiative (YD9110002053 and YD9110002121); the Research Funds of Centre for Leading Medicine and Advanced Technologies of IHM (2023IHM01070 and 2023IHM01074); and the Xizang Natural Science Foundation of Xizang Autonomous Region (XZ202601ZR0060).

## Declaration of competing interests

The authors declare that they have no known competing financial interests or personal relationships that could have appeared to influence the work reported in this paper.

## References

[1] Oliveira T.C., Gomes M.S., Gomes A.C. (2020). The crossroads between infection and bone loss. Microorganisms.

[2] Obst U., Marten S.M., Niessner C., Hartwig E. (2011). Bacterial DNA from orthopedic implants after routine removal. Int J Artif Organs.

[3] Kaushik A., Kest H., Sood M., Steussy B.W., Thieman C., Gupta S. (2024). Biofilm-producing methicillin-resistant Staphylococcus aureus (MRSA) infections in humans: clinical implications and management. Pathogens.

[4] Chen Y., Liu Z., Lin Z., Lu M., Fu Y., Liu G. (2023). The effect of Staphylococcus aureus on innate and adaptive immunity and potential immunotherapy for *S. aureus*-induced osteomyelitis. Front Immunol.

[5] Wen Q., Gu F., Sui Z., Su Z., Yu T. (2020). The process of osteoblastic infection by Staphylococcus aureus. Int J Med Sci.

[6] Qin L., Hu N., Zhang Y., Yang J., Zhao L., Zhang X. (2024). Antibody-antibiotic conjugate targeted therapy for orthopedic implant-associated intracellular S. aureus infections. J Adv Res.

[7] Nasser A., Dallal M.M.S., Jahanbakhshi S., Azimi T., Nikouei L. (2022). Staphylococcus aureus: biofilm formation and strategies against it. Curr Pharm Biotechnol.

[8] Masters E.A., Ricciardi B.F., Bentley K.L.M., Moriarty T.F., Schwarz E.M., Muthukrishnan G. (2022). Skeletal infections: microbial pathogenesis, immunity and clinical management. Nat Rev Microbiol.

[9] Campoccia D., Mirzaei R., Montanaro L., Arciola C.R. (2019). Hijacking of immune defences by biofilms: a multifront strategy. Biofouling.

[10] Gao L., Tang Z., Zhang Z., Wei D., Wang J. (2025). Transcription factor FOS promotes ferroptosis and inflammation in S. aureus-infected osteomyelitis via EIF5A. J Orthop Surg Res.

[11] Isojima T., Crimeen-Irwin B., McGregor N.E., Chai R.C., Poulton I.J., Walker E.C. (2025). Bone marrow neutrophil progenitors suppress osteoclast formation in murine cortical and trabecular bone. Blood.

[12] Dapunt U., Giese T., Maurer S., Stegmaier S., Prior B., Hansch G.M. (2015). Neutrophil-derived MRP-14 is up-regulated in infectious osteomyelitis and stimulates osteoclast generation. J Leukoc Biol.

[13] Scozzi D., Liao F., Krupnick A.S., Kreisel D., Gelman A.E. (2022). The role of neutrophil extracellular traps in acute lung injury. Front Immunol.

[14] Peng S.L. (2006). Neutrophil apoptosis in autoimmunity. J Mol Med (Berl).

[15] Li J., Ye L.J., Dai Y.W., Wang H.W., Gao J., Shen Y.H. (2024). Single-cell analysis reveals a unique microenvironment in peri-implantitis. J Clin Periodontol.

[16] Akaraphanth M., Nordgren T.M., Gries C.M. (2024). Cxcr2 perturbation promotes Staphylococcus aureus implant-associated infection. J Med Microbiol.

[17] Ramesh R., Rekha N.D., Gopal S. (2025). Pseudomonas aeruginosa biofilm: treatment strategies to combat infection. Arch Microbiol.

[18] Labow R.S., Meek E., Santerre J.P. (2001). Neutrophil-mediated biodegradation of medical implant materials. J Biomed Mater Res.

[19] Zhou Y., Doerschuk C.M., Anderson J.M., Marchant R.E. (2004). Biomaterial surface-dependent neutrophil mobility. J Biomed Mater Res.

[20] Liu H., Xu D., Ma Y., Qian J., Yang Y., Yu B. (2022). Mechanisms of hierarchical topographies tuning bacteria and cell biological responses to the surfaces of pure titanium and cu-bearing titanium alloy. ACS Appl Mater Interfaces.

[21] Okshevsky M., Meyer R.L. (2015). The role of extracellular DNA in the establishment, maintenance and perpetuation of bacterial biofilms. Crit Rev Microbiol.

[22] Gonzalez C.D., Ledo C., Cela E., Stella I., Xu C., Ojeda D.S. (2019). The good side of inflammation: staphylococcus aureus proteins spa and sbi contribute to proper abscess formation and wound healing during skin and soft tissue infections. Biochim Biophys Acta Mol Basis Dis.

[23] Ng L.G., Kwok I. (2025). Transcending life and death: the ultimate cargo of aged neutrophils. Cell.

[24] Wang Y., Shi Y., Shao Y., Lu X., Zhang H., Miao C. (2024). S100a8/a9(hi) neutrophils induce mitochondrial dysfunction and PANoptosis in endothelial cells via mitochondrial complex I deficiency during sepsis. Cell Death Dis.

[25] Milot E., Filep J.G. (2011). Regulation of neutrophil survival/apoptosis by Mcl-1. ScientificWorldJournal.

[26] Meyle E., Brenner-Weiss G., Obst U., Prior B., Hansch G.M. (2012). Immune defense against S. epidermidis biofilms: components of the extracellular polymeric substance activate distinct bactericidal mechanisms of phagocytic cells. Int J Artif Organs.

[27] Wang Y., Zhang L.F., Zhang J.J., Yu S.S., Li W.L., Zhou T.J. (2025). Spontaneous inflammation resolution inspired nanoparticles promote neutrophil apoptosis and macrophage efferocytosis for acute respiratory distress syndrome treatment. Adv Healthcare Mater.

[28] Filep J.G., Ariel A. (2020). Neutrophil heterogeneity and fate in inflamed tissues: implications for the resolution of inflammation. Am J Physiol Cell Physiol.

[29] Peng S., Gao J., Stojkov D., Yousefi S., Simon H.U. (2023). Established and emerging roles for mitochondria in neutrophils. Immunol Rev.

[30] Perez-Figueroa E., Alvarez-Carrasco P., Ortega E., Maldonado-Bernal C. (2021). Neutrophils: many ways to die. Front Immunol.

[31] Yamada K.J., Kielian T. (2019). Biofilm-leukocyte cross-talk: impact on immune polarization and immunometabolism. J Innate Immun.

[32] Wang Z., Li M., Li W., He L., Wang L., Cai K. (2025). Hybrid outer membrane vesicles with genetically engineering for treatment of implant-associated infections and relapse prevention through host immunomodulation. Adv Sci (Weinh).

[33] Liu Z., Jin G., Zhi Y., Ge S., Wang D., Li J. (2025). Trends in immune cell profiles of osteomyelitis: a clinical study supported by Mendelian randomization analysis. Front Med.

[34] Li T., Nan S., Xie H., Song Y., Chai W. (2026). Single-cell RNA sequencing analysis reveals the molecular mechanisms of neutrophil dysfunction in chronic bone infection. Curr Med Chem.

[35] Yuan J., Ofengeim D. (2024). A guide to cell death pathways. Nat Rev Mol Cell Biol.

[36] de Vor L., Rooijakkers S.H.M., van Strijp J.A.G. (2020). Staphylococci evade the innate immune response by disarming neutrophils and forming biofilms. FEBS Lett.

[37] Li J., Li H., Bi S., Sun Y., Gu F., Yu T. (2024). Shock wave assisted intracellular delivery of antibiotics against bone infection with Staphylococcus aureus via p2x7 receptors. J Orthop Transl.

[38] Feng T., Chen P., Lin F. (2025). Identification and analysis of diverse cell death patterns in osteomyelitis via microarray-based transcriptome profiling and clinical data. Front Immunol.

[39] Ma L., Cheng Y., Feng X., Zhang X., Lei J., Wang H. (2024). A janus-ros healing system promoting infectious bone regeneration via sono-epigenetic modulation. Adv Mater.

[40] Glennon-Alty L., Moots R.J., Edwards S.W., Wright H.L. (2021). Type I interferon regulates cytokine-delayed neutrophil apoptosis, reactive oxygen species production and chemokine expression. Clin Exp Immunol.

[41] Zhou W., Cao X., Xu Q., Qu J., Sun Y. (2023). The double-edged role of neutrophil heterogeneity in inflammatory diseases and cancers. MedComm (2020).

[42] Sipprell S.E., Krueger Q.A., Mills E.L., Marriott I., Johnson M.B. (2025). Substance p augments chemokine production by Staphylococcus aureus infected murine osteoclasts. Inflammation.

[43] Liu J., Wei T., Quan L., Dou M., Yue J., Yuan P. (2025). Identification and multi-dimensional validation of mitochondrial permeability transition-driven necrosis-related model to assess the prognosis and immunotherapy value in breast cancer. Eur J Med Res.

[44] Zi S.F., Wu X.J., Tang Y., Liang Y.P., Liu X., Wang L. (2024). Endothelial cell‐derived extracellular vesicles promote aberrant neutrophil trafficking and subsequent remote lung injury. Adv Sci (Weinh).

[45] Wagner C., Obst U., Hänsch G.M. (2005). Implant-associated posttraumatic osteomyelitis: collateral damage by local host defense. Int J Artif Organs.

[46] Kalogeris T., Baines C.P., Krenz M., Korthuis R.J. (2012). Cell biology of ischemia/reperfusion injury. Int Rev Cell Mol Biol.

[47] Hornstein T., Spannbrucker T., Unfried K. (2025). Combustion-derived carbon nanoparticles cause delayed apoptosis in neutrophil-like HL-60 cells in vitro and in primed human neutrophilic granulocytes ex vivo. Part Fibre Toxicol.

[48] Hofstee M.I., Riool M., Terjajevs I., Thompson K., Stoddart M.J., Richards R.G. (2020). Three-dimensional in vitro Staphylococcus aureus abscess communities display antibiotic tolerance and protection from neutrophil clearance. Infect Immun.

[49] Garrido-Trigo A., Corraliza A.M., Veny M., Dotti I., Melon-Ardanaz E., Rill A. (2023). Macrophage and neutrophil heterogeneity at single-cell spatial resolution in human inflammatory bowel disease. Nat Commun.

[50] Peng H., Wu X., Liu S., He M., Tang C., Wen Y. (2023). Cellular dynamics in tumour microenvironment along with lung cancer progression underscore spatial and evolutionary heterogeneity of neutrophil. Clin Transl Med.

[51] Xue R., Zhang Q., Cao Q., Kong R., Xiang X., Liu H. (2022). Liver tumour immune microenvironment subtypes and neutrophil heterogeneity. Nature.

[52] Zhu Z., Zhou S., Li S., Gong S., Zhang Q. (2024). Neutrophil extracellular traps in wound healing. Trends Pharmacol Sci.

[53] Liu Y., Xiang C., Que Z., Li C., Wang W., Yin L. (2023). Neutrophil heterogeneity and aging: implications for covid-19 and wound healing. Front Immunol.

[54] Lin H., Liao Y., Chen Z., Chen Y., Chen Y., Wang Y. (2025). Mechanistic roles of neutrophil heterogeneity in tumour pathogenesis. Front Immunol.

[55] O'Neil L.J., Oliveira C.B., Wang X., Navarrete M., Barrera-Vargas A., Merayo-Chalico J. (2023). Neutrophil extracellular trap-associated carbamylation and histones trigger osteoclast formation in rheumatoid arthritis. Ann Rheum Dis.

[56] Chen Y., Li C., Jia J., Jiang Y., Zhang P., Cheng C. (2025). Cox-2 inhibition as a therapeutic strategy for bone loss in Staphylococcus aureus osteomyelitis. Mol Med.

[57] Kim C., Kim H., Sim W.S., Jung M., Hong J., Moon S. (2024). Spatiotemporal control of neutrophil fate to tune inflammation and repair for myocardial infarction therapy. Nat Commun.

[58] Nourshargh S., Renshaw S.A., Imhof B.A. (2016). Reverse migration of neutrophils: where, when, how, and why?. Trends Immunol.

[59] de Oliveira S., Rosowski E.E., Huttenlocher A. (2016). Neutrophil migration in infection and wound repair: going forward in reverse. Nat Rev Immunol.

[60] Amulic B., Cazalet C., Hayes G.L., Metzler K.D., Zychlinsky A. (2012). Neutrophil function: from mechanisms to disease. Annu Rev Immunol.

[61] Wright D.G. (1988). Human neutrophil degranulation. Methods Enzymol.

[62] Silvestre-Roig C., Hidalgo A., Soehnlein O. (2016). Neutrophil heterogeneity: implications for homeostasis and pathogenesis. Blood.

[63] Xie X., Shi Q., Wu P., Zhang X., Kambara H., Su J. (2020). Single-cell transcriptome profiling reveals neutrophil heterogeneity in homeostasis and infection. Nat Immunol.

[64] Montaldo E., Lusito E., Bianchessi V., Caronni N., Scala S., Basso-Ricci L. (2022). Cellular and transcriptional dynamics of human neutrophils at steady state and upon stress. Nat Immunol.

[65] Zhang F., Xia Y., Su J., Quan F., Zhou H., Li Q. (2024). Neutrophil diversity and function in health and disease. Signal Transduct Target Ther.

[66] Silvestre-Roig C., Fridlender Z.G., Glogauer M., Scapini P. (2019). Neutrophil diversity in health and disease. Trends Immunol.

[67] Gysemans C., Beya M., Pedace E., Mathieu C. (2025). Exploring neutrophil heterogeneity and plasticity in health and disease. Biomedicines.

[68] Grieshaber-Bouyer R., Radtke F.A., Cunin P., Stifano G., Levescot A., Vijaykumar B. (2021). The neutrotime transcriptional signature defines a single continuum of neutrophils across biological compartments. Nat Commun.

[69] Cassatella M.A., Ostberg N.K., Tamassia N., Soehnlein O. (2019). Biological roles of neutrophil-derived granule proteins and cytokines. Trends Immunol.

[70] Quail D.F., Amulic B., Aziz M., Barnes B.J., Eruslanov E., Fridlender Z.G. (2022). Neutrophil phenotypes and functions in cancer: a consensus statement. J Exp Med.

[71] Lin Z.Q., Chen D., Zhang P.D., Luo J.L., Chen S.Y., Gu S.P. (2025). Multi-omics reveal neutrophil heterogeneity in sepsis. Int J Mol Med.

[72] Bashant K.R., Aponte A.M., Randazzo D., Rezvan Sangsari P., Wood A.J., Bibby J.A. (2021). Proteomic, biomechanical and functional analyses define neutrophil heterogeneity in systemic lupus erythematosus. Ann Rheum Dis.

[73] Sui D., Liu F., Zheng L., Yu S., Ding Z., Ren X. (2026). Disease-associated neutrophil heterogeneity in cancer therapy: implications for precision immunotherapy. Crit Rev Oncol Hematol.

[74] Liu S., Zhou Y., Li G., Zhu B., Wu F., Zhou J. (2025). Plaur(+) neutrophils drive anti-pd-1 therapy resistance in patients with hepatocellular carcinoma by shaping an immunosuppressive microenvironment. Adv Sci (Weinh).

[75] Zhou G., Yu L., Fang L., Yang W., Yu T., Miao Y. (2018). Cd177(+) neutrophils as functionally activated neutrophils negatively regulate ibd. Gut.

[76] Ng L.G., Ballesteros I., Cassatella M.A., Egeblad M., Fridlender Z.G., Gabrilovich D. (2025). From complexity to consensus: a roadmap for neutrophil classification. Immunity.

[77] Feng Y., Liu G., Li H., Cheng L. (2025). Target neutrophil heterogeneity and plasticity in cancer. J Hematol Oncol.

[78] Mouillot P., Witko-Sarsat V., Wislez M. (2022). [neutrophil plasticity: a new key in the understanding of onco-immunology]. Rev Mal Respir.

[79] Morrison T., Watts E.R., Sadiku P., Walmsley S.R. (2023). The emerging role for metabolism in fueling neutrophilic inflammation. Immunol Rev.

[80] Jin H., Aziz M., Murao A., Kobritz M., Shih A.J., Adelson R.P. (2023). Antigen-presenting aged neutrophils induce CD4⁺ T cells to exacerbate inflammation in sepsis. J Clin Investig.

[81] Jaboury S., Wang K., O'Sullivan K.M., Ooi J.D., Ho G.Y. (2023). Netosis as an oncologic therapeutic target: a mini review. Front Immunol.

[82] Chan L., Wood G.A., Wootton S.K., Bridle B.W., Karimi K. (2023). Neutrophils in dendritic cell-based cancer vaccination: the potential roles of neutrophil extracellular trap formation. Int J Mol Sci.

[83] Mihlan M., Wissmann S., Gavrilov A., Kaltenbach L., Britz M., Franke K. (2024). Neutrophil trapping and nexocytosis, mast cell-mediated processes for inflammatory signal relay. Cell.

[84] Yao Y., Yin Y., Shuai F., Lam W., Zhou T., Xie Y. (2025). M2 macrophage‐derived extracellular vesicles reprogram immature neutrophils into anxa1hi neutrophils to enhance inflamed bone regeneration. Adv Sci (Weinh).

[85] Barth N.D., Vendrell M., Dorward D.A., Rossi A.G., Dransfield I. (2020). Assessment of neutrophil apoptosis. Methods Mol Biol.

[86] Lagunas-Rangel F.A. (2025). Neutrophil-to-lymphocyte ratio in aging: trends and clinical implications. Exp Gerontol.

[87] Kalafati L., Hatzioannou A., Hajishengallis G., Chavakis T. (2023). The role of neutrophils in trained immunity. Immunol Rev.

[88] Ng L.G., Ostuni R., Hidalgo A. (2019). Heterogeneity of neutrophils. Nat Rev Immunol.

[89] Metzemaekers M., Malengier-Devlies B., Gouwy M., De Somer L., Cunha F.Q., Opdenakker G. (2023). Fast and furious: the neutrophil and its armamentarium in health and disease. Med Res Rev.

[90] Arciola C.R., Ravaioli S., Mirzaei R., Dolzani P., Montanaro L., Daglia M. (2023). Biofilms in periprosthetic orthopedic infections seen through the eyes of neutrophils: how can we help neutrophils?. Int J Mol Sci.

[91] Brostjan C., Oehler R. (2020). The role of neutrophil death in chronic inflammation and cancer. Cell Death Discov.

[92] de Jesus Gonzalez-Contreras F., Zarate X. (2022). Neutrophil extracellular traps: modulation mechanisms by pathogens. Cell Immunol.

[93] Hampson P., Hazeldine J., Lord J.M. (2013). Neutrophil apoptosis and its induction as a potential treatment for chronic inflammatory disease. Curr Opin Hematol.

[94] Wang H., Mu N., He Y., Zhang X., Lei J., Yang C. (2023). Ultrasound-controlled mxene-based schottky heterojunction improves anti-infection and osteogenesis properties. Theranostics.

[95] Chinipardaz Z., Zhong J.M., Yang S. (2022). Regulation of ll-37 in bone and periodontium regeneration. Life.

[96] Angeletti A., Volpi S., Bruschi M., Lugani F., Vaglio A., Prunotto M. (2021). Neutrophil extracellular traps-dnase balance and autoimmunity. Cells.

[97] Fu J., Zhu W., Liu X., Liang C., Zheng Y., Li Z. (2021). Self-activating anti-infection implant. Nat Commun.

[98] Hidalgo A., Casanova-Acebes M. (2021). Dimensions of neutrophil life and fate. Semin Immunol.

[99] Dai T., Wen Y., Li Q., Wang H., Liao H., Pu Y. (2025). Synovial advanced glycosylation end products aggravate periprosthetic infection in diabetes by upregulating Staphylococcus aureus rnaiii. J Orthop Transl.

[100] Smith W.B., Gamble J.R., Clark-Lewis I., Vadas M.A. (1991). Interleukin-8 induces neutrophil transendothelial migration. Immunology.

[101] Kranjec C., Morales Angeles D., Torrissen Marli M., Fernandez L., Garcia P., Kjos M. (2021). Staphylococcal biofilms: challenges and novel therapeutic perspectives. Antibiotics (Basel).

[102] Campoccia D., Ravaioli S., Mirzaei R., Bua G., Daglia M., Arciola C.R. (2023). Interactions of neutrophils with the polymeric molecular components of the biofilm matrix in the context of implant-associated bone and joint infections. Int J Mol Sci.

[103] Khatib-Massalha E., Bhattacharya S., Massalha H., Biram A., Golan K., Kollet O. (2020). Lactate released by inflammatory bone marrow neutrophils induces their mobilization via endothelial gpr81 signaling. Nat Commun.

[104] Akers K.S., Cardile A.P., Wenke J.C., Murray C.K. (2015). Biofilm formation by clinical isolates and its relevance to clinical infections. Adv Exp Med Biol.

[105] Panlilio H., Rice C.V. (2021). The role of extracellular DNA in the formation, architecture, stability, and treatment of bacterial biofilms. Biotechnol Bioeng.

[106] Dapunt U., Hänsch G.M., Arciola C.R. (2016). Innate immune response in implant-associated infections: neutrophils against biofilms. Materials.

[107] Arciola C.R., Campoccia D., Montanaro L. (2018). Implant infections: adhesion, biofilm formation and immune evasion. Nat Rev Microbiol.

[108] Dapunt U., Gaida M.M., Meyle E., Prior B., Hänsch G.M. (2016). Activation of phagocytic cells by Staphylococcus epidermidis biofilms: effects of extracellular matrix proteins and the bacterial stress protein GroEL on netosis and MRP-14 release. Pathog Dis.

[109] Maurer S., Fouchard P., Meyle E., Prior B., Hänsch G.M., Dapunt U. (2015). Activation of neutrophils by the extracellular polymeric substance of *S. epidermidis* biofilms is mediated by the bacterial heat shock protein GroEL. J Biotechnol Biomater.

[110] Sekheri M., Othman A., Filep J.G. (2021). Beta2 integrin regulation of neutrophil functional plasticity and fate in the resolution of inflammation. Front Immunol.

[111] Zhu Y., Xia X., He Q., Xiao Q.A., Wang D., Huang M. (2023). Diabetes-associated neutrophil netosis: pathogenesis and interventional target of diabetic complications. Front Endocrinol.

[112] Sambataro M., Sambado L., Colardo M., Furlan A., Stefani P.M., Durante E. (2025). Neuroinflammation and osteomyelitis in adults with type 2 diabetes mellitus and peripheral neuropathy without and with foot lesions. What comes first?. J Diabetes Complications.

[113] Zhang Y., Li B., Liu C., Huang T., Rupp M., Brochhausen C. (2026). Functional biomaterials for the treatment of periprosthetic joint infection: a systematic review of preclinical animal studies. J Orthop Transl.

[114] Gaida M.M., Mayer B., Stegmaier S., Schirmacher P., Wagner C., Hänsch G.M. (2012). Polymorphonuclear neutrophils in osteomyelitis: link to osteoclast generation and bone resorption. Eur J Inflamm.

[115] Dapunt U., Giese T., Lasitschka F., Lehner B., Ewerbeck V., Hänsch G.M. (2013). Osteoclast generation and cytokine profile at prosthetic interfaces: a study on tissue of patients with aseptic loosening or implant-associated infections. J Biomed Mater Res.

[116] Wang K., Hampson P., Hazeldine J., Krystof V., Strnad M., Pechan P. (2012). Cyclin-dependent kinase 9 activity regulates neutrophil spontaneous apoptosis. PLoS One.

[117] Liu X., Ou X., Zhang T., Li X., Qiao Q., Jia L. (2024). In situ neutrophil apoptosis and macrophage efferocytosis mediated by glycyrrhiza protein nanoparticles for acute inflammation therapy. J Contr Release.

[118] Strieter R.M., Kasahara K., Allen R.M., Standiford T.J., Rolfe M.W., Becker F.S. (1992). Cytokine-induced neutrophil-derived interleukin-8. Am J Pathol.

[119] Ling S., Xu J.W. (2024). Phenotypes and functions of "aged" neutrophils in cardiovascular diseases. Biomed Pharmacother.

[120] Carmona-Rivera C., Kaplan M.J., O'Neil L.J. (2024). Neutrophils in inflammatory bone diseases. Curr Osteoporos Rep.

[121] Guo C.L., Wang C.S., Wang Z.C., Liu F.F., Liu L., Yang Y. (2024). Granzyme k(+)cd8(+) t cells interact with fibroblasts to promote neutrophilic inflammation in nasal polyps. Nat Commun.

[122] Sundqvist M., Christenson K., Wekell P., Bjornsdottir H., Dahlstrand Rudin A., Sanchez Klose F.P. (2023). Severe chronic non-bacterial osteomyelitis in combination with total MPO deficiency and responsiveness to TNFα inhibition. Front Immunol.

[123] Zhu M., Barbas A.S., Lin L., Scheuermann U., Bishawi M., Brennan T.V. (2018). Mitochondria released by apoptotic cell death initiate innate immune responses. ImmunoHorizons.

[124] Marshall J.C., Malam Z., Jia S. (2007). Modulating neutrophil apoptosis. Novartis Found Symp.

[125] Martin-Nalda A., Roca I., Fontecha C.G., Fernandez-Polo A., Barber I., Martinez-Gallo M. (2016). Chronic recurrent multifocal osteomyelitis and thalidomide in chronic granulomatous disease. Pediatrics.

[126] Adrover J.M., Del Fresno C., Crainiciuc G., Cuartero M.I., Casanova-Acebes M., Weiss L.A. (2019). A neutrophil timer coordinates immune defense and vascular protection. Immunity.

[127] Webb P.R., Wang K.-Q., Scheel-Toellner D., Pongracz J., Salmon M., Lord J.M. (2000). Regulation of neutrophil apoptosis: a role for protein kinase c and phosphatidylinositol-3-kinase. J Leukoc Biol.

[128] O'Neill A., Gately M.C., Doyle B., Fitzpatrick J.M., Watson R.W.G. (2004). Gene profiling of in vitro and in vivo models of delayed neutrophil apoptosis: a common pathway?. J Leukoc Biol.

[129] Granata V., Possetti V., Parente R., Bottazzi B., Inforzato A., Sobacchi C. (2022). The osteoblast secretome in Staphylococcus aureus osteomyelitis. Front Immunol.

[130] Simon H.-U. (2003). Neutrophil apoptosis pathways and their modifications in inflammation. Immunol Rev.

[131] Bai M., Jin Y., Jin Z., Xie Y., Chen J., Zhong Q. (2025). Distinct immunophenotypic profiles and neutrophil heterogeneity in colorectal cancer. Cancer Lett.

[132] Asensi V., Valle E., Meana A., Fierer J., Celada A., Alvarez V. (2004). In vivo interleukin-6 protects neutrophils from apoptosis in osteomyelitis. Infect Immun.

[133] Almeida-Junior L.A., de Carvalho M.S., Almeida L.K.Y., Silva-Sousa A.C., Sousa-Neto M.D., Silva R.A.B. (2023). Tnf-alpha-tnfr1 signaling mediates inflammation and bone resorption in apical periodontitis. J Endod.

[134] Hedrick C.C., Malanchi I. (2022). Neutrophils in cancer: heterogeneous and multifaceted. Nat Rev Immunol.

[135] Zhan Y., Ling Y., Deng Q., Qiu Y., Shen J., Lai H. (2022). Hmgb1-mediated neutrophil extracellular trap formation exacerbates intestinal ischemia/reperfusion-induced acute lung injury. J Immunol.

[136] Surmiak M., Hubalewska-Mazgaj M., Wawrzycka-Adamczyk K., Musial J., Sanak M. (2020). Delayed neutrophil apoptosis in granulomatosis with polyangiitis: dysregulation of neutrophil gene signature and circulating apoptosis-related proteins. Scand J Rheumatol.

[137] Lentini G., Fama A., De Gaetano G.V., Coppolino F., Mahjoub A.K., Ryan L. (2023). Caspase-8 inhibition improves the outcome of bacterial infections in mice by promoting neutrophil activation. Cell Rep Med.

[138] Sabbatini M., Magnelli V., Reno F. (2021). Netosis in wound healing: when enough is enough. Cells.

[139] Xiang J., Cui M., Zhang Y. (2025). Neutrophil extracellular traps and neutrophilic asthma. Respir Med.

[140] Gao W., Wang X., Zhou Y., Wang X., Yu Y. (2022). Autophagy, ferroptosis, pyroptosis, and necroptosis in tumor immunotherapy. Signal Transduct Target Ther.

[141] Lee J., Jin B.R., Cho J. (2025). Spatiotemporal regulation of neutrophil heterogeneity in health and disease. Hum Mol Genet.

[142] Zhu W., Mei J., Zhang X., Zhou J., Xu D., Su Z. (2022). Photothermal nanozyme-based microneedle patch against refractory bacterial biofilm infection via iron-actuated janus ion therapy. Adv Mater.

[143] Markowska A., Tarnacka B. (2025). Idiopathic inflammatory myopathy-molecular mechanisms underlying its pathogenesis and physical therapy effects. Int J Mol Sci.

[144] Meyers S., Crescente M., Verhamme P., Martinod K. (2022). Staphylococcus aureus and neutrophil extracellular traps: the master manipulator meets its match in immunothrombosis. Arterioscler Thromb Vasc Biol.

[145] Zhou J., Mei J., Liu Q., Xu D., Wang X., Zhang X. (2023). Spatiotemporal on–off immunomodulatory hydrogel targeting NLRP3 inflammasome for the treatment of biofilm‐infected diabetic wounds. Adv Funct Mater.

[146] Mei J., Zhou J., Kong L., Dai Y., Zhang X., Song W. (2022). An injectable photo-cross-linking silk hydrogel system augments diabetic wound healing in orthopaedic surgery through spatiotemporal immunomodulation. J Nanobiotechnol.

[147] Beagan M.L.C., Dreyer C.H., Jensen L.K., Jensen H.E., Andersen T.E., Overgaard S. (2024). The potential of sheep in preclinical models for bone infection research – a systematic review. J Orthop Transl.

[148] Liu H., Fan T., Yuan R., Lu S., Sun D., Huan Y. (2025). Establishment of a clinically relevant beagle model for periprosthetic joint infection with 3D-printed prostheses and multimodal evaluation. J Orthop Transl.

[149] Thierry A.R., Salmon D. (2024). Inflammation-, immunothrombosis,- and autoimmune-feedback loops may lead to persistent neutrophil self-stimulation in long COVID. J Med Virol.

[150] Zhu W., Mei J., Xu D., Wang J., Zhou J., Hu X. (2025). Therapeutic in situ nano-vaccine for combating medical implant-related biofilm infection. Cell Biomaterials.

[151] Cao Z., Xu R., Zheng W., Ma L., He Y., Chen T. (2025). Ultrasound-activated selenium nanocarrier: bactericidal enhancement and osseointegration promotion for implant-associated infections. Adv Healthcare Mater.

[152] Yang D., Shu C., Xu C., Zan X., Wei S., Wang C. (2025). Bacterial capture-killing capsules with remodeling bone immune microenvironment for the effective treatment of osteomyelitis. Adv Sci (Weinh).

[153] Li Y., Xie X., Ding C., Zhang S., Hu L., Mi B. (2025). Immunomodulatory biomaterials for bone and soft tissue chronic inflammation diseases. Small Sci.

[154] Meier A., Sakoulas G., Nizet V., Ulloa E.R. (2024). Neutrophil extracellular traps: an emerging therapeutic target to improve infectious disease outcomes. J Infect Dis.

[155] von Locquenghien M., Zwicky P., Xie K., Jaitin D.A., Sheban F., Yalin A. (2025). Macrophage-targeted immunocytokine leverages myeloid, T, and NK cell synergy for cancer immunotherapy. Cell.

[156] Lee C.W., Lin Z.C., Chiang Y.C., Li S.Y., Ciou J.J., Liu K.W. (2023). AuAg nanocomposites suppress biofilm-induced inflammation in human osteoblasts. Nanotechnology.

[157] Su Z., Kong L., Mei J., Li Q., Qian Z., Ma Y. (2023). Enzymatic bionanocatalysts for combating peri-implant biofilm infections by specific heat-amplified chemodynamic therapy and innate immunomodulation. Drug Resist Updates.

[158] Su Z., Xu D., Hu X., Zhu W., Kong L., Qian Z. (2024). Biodegradable oxygen-evolving metalloantibiotics for spatiotemporal sono-metalloimmunotherapy against orthopaedic biofilm infections. Nat Commun.

[159] Mei J., Xu D., Wang L., Kong L., Liu Q., Li Q. (2023). Biofilm microenvironment-responsive self-assembly nanoreactors for all-stage biofilm associated infection through bacterial cuproptosis-like death and macrophage re-rousing. Adv Mater.

[160] Wang Z., Zhang X., Liu Q., Hu X., Mei J., Zhou J. (2024). Balancing bioresponsive biofilm eradication and guided tissue repair via pro-efferocytosis and bidirectional pyroptosis regulation during implant surgery. ACS Nano.

[161] Zhao F., Gao A., Liao Q., Li Y., Ullah I., Zhao Y. (2024). Balancing the anti‐bacterial and pro‐osteogenic properties of Ti‐based implants by partial conversion of ZnO nanorods into hybrid zinc phosphate nanostructures. Adv Funct Mater.

[162] Cai Y., Liang J., Chen X., Zhang G., Jing Z., Zhang R. (2023). Synovial fluid neutrophil extracellular traps could improve the diagnosis of periprosthetic joint infection. Bone Joint Res.

